# A highly potent anti-VISTA antibody KVA12123 - a new immune checkpoint inhibitor and a promising therapy against poorly immunogenic tumors

**DOI:** 10.3389/fimmu.2023.1311658

**Published:** 2023-12-12

**Authors:** Shawn Iadonato, Yulia Ovechkina, Kurt Lustig, Jessica Cross, Nathan Eyde, Emily Frazier, Neda Kabi, Chen Katz, Remington Lance, David Peckham, Shaarwari Sridhar, Carla Talbaux, Isabelle Tihista, Mei Xu, Thierry Guillaudeux

**Affiliations:** Kineta Inc., Seattle, WA, United States

**Keywords:** Vista, PD-1H, B7-H5, immune checkpoint inhibitor, immunotherapy, PD-1 combination therapy, poorly immunogenic tumors, tumor microenvironment immunosuppression

## Abstract

**Background:**

Immune checkpoint therapies have led to significant breakthroughs in cancer patient treatment in recent years. However, their efficiency is variable, and resistance to immunotherapies is common. VISTA is an immune-suppressive checkpoint inhibitor of T cell response belonging to the B7 family and a promising novel therapeutic target. VISTA is expressed in the immuno-suppressive tumor microenvironment, primarily by myeloid lineage cells, and its genetic knockout or antibody blockade restores an efficient antitumor immune response.

**Methods:**

Fully human monoclonal antibodies directed against VISTA were produced after immunizing humanized Trianni mice and sorting and sequencing natively-linked B cell scFv repertoires. Anti-VISTA antibodies were evaluated for specificity, cross-reactivity, monocyte and T cell activation, Fc-effector functions, and antitumor efficacy using *in vitro* and *in vivo* models to select the KVA12123 antibody lead candidate. The pharmacokinetics and safety profiles of KVA12123 were evaluated in cynomolgus monkeys.

**Results:**

Here, we report the development of a clinical candidate anti-VISTA monoclonal antibody, KVA12123. KVA12123 showed high affinity binding to VISTA through a unique epitope distinct from other clinical-stage anti-VISTA monoclonal antibodies. This clinical candidate demonstrated high specificity against VISTA with no cross-reactivity detected against other members of the B7 family. KVA12123 blocked VISTA binding to its binding partners. KVA12123 induced T cell activation and demonstrated NK-mediated monocyte activation. KVA12123 treatment mediated strong single-agent antitumor activity in several syngeneic tumor models and showed enhanced efficacy in combination with anti-PD-1 treatment. This clinical candidate was engineered to improve its pharmacokinetic characteristics and reduce Fc-effector functions. It was well-tolerated in preclinical toxicology studies in cynomolgus monkeys, where hematology, clinical chemistry evaluations, and clinical observations revealed no indicators of toxicity. No cytokines associated with cytokine release syndrome were elevated.

**Conclusion:**

These results establish that KVA12123 is a promising drug candidate with a distinct but complementary mechanism of action of the first generation of immune checkpoint inhibitors. This antibody is currently evaluated alone and in combination with pembrolizumab in a Phase 1/2 open-label clinical trial in patients with advanced solid tumors.

## 1 Introduction

The development of first-generation immune checkpoint therapies targeting PD-(L)1 or CTLA-4 led to efficient anti-tumor T cell responses, resulting in durable, long-lasting clinical outcomes, but only in a fraction of cancer patients (1–3). Novel therapeutics are needed to help overcome resistance and improve treatment in non-responders or in patients who relapse from these therapies. Cancer cells often utilize immunosuppressive strategies in the tumor microenvironment (TME) to continue to proliferate. VISTA (V-domain Ig suppressor of T cell activation) is a key driver of immuno-suppression. It plays an important role in maintaining immune tolerance in a healthy state but allows tumors to avoid an effective immune response (4–8). VISTA is a type I transmembrane immunomodulatory glycoprotein of the B7 family, also known as PD-1H (programmed death-1 homolog), B7-H5, PD-1H, Gi24, Dies1, SISP1, and DD1α. VISTA shares 25% of its protein sequence identity with its closest homolog, PD-L1, but with unique structural features, expression patterns, and functions. VISTA is mainly expressed on circulating and intra-tumoral myeloid cells as well as Treg and NK cells (5, 8). VISTA expression is not restricted to the cell surface but is also detected in the early endosomes of myeloid cells, where it colocalizes with markers for early endosomes (EEA-1) and recycling endosomes (Rab-11), suggesting that VISTA is actively recycled back to the extracellular membrane (9). It has been demonstrated that VISTA inhibits T cell activation and modulates the migration and activation of macrophages and myeloid-derived suppressor cells (MDSCs) in the TME (5, 8, 10, 11). VISTA is highly expressed in tumors that are poorly infiltrated by T cells, also described as cold tumors, and high expression of VISTA has been associated with poor overall survival in different tumor indications like melanoma, pancreatic or prostate cancers (12–15). VISTA genetic knockout or blocking VISTA with monoclonal antibodies (mAbs) in mice led to tumor-specific effector T cell activation, reduced Treg function, and enhanced myeloid-mediated inflammatory responses. In cancer patients, VISTA is also a potential mediator of resistance to anti-CTLA-4 and anti-PD(L)1 therapies, where its overexpression has been associated with patients’ relapses (16, 17), making VISTA an attractive target for combination with other anti-cancer immunotherapies.

Here, we describe the discovery, characterization, and preclinical development of KVA12123, an antagonist anti-human VISTA monoclonal antibody (mAb). Our clinical candidate, KVA12123, is a fully human IgG1-kappa mAb engineered to increase its half-life and reduce Fc-mediated immune effector functions. KVA12123 binds human VISTA at neutral and acidic pH, blocking its interaction with four known VISTA binding partners: LRIG1, VSIG3, VSIG8, and PSGL-1. KVA12123 mAbs recognize the cynomolgus monkey VISTA with a similar binding affinity to human VISTA. Mutagenesis analyses performed on the human VISTA extracellular domain (hVISTA-ECD) demonstrated that residues Y37, R54, V117, and R127 are the critical amino acids responsible for KVA12123 epitope binding on VISTA. KVA12123 mAbs showed strong antitumor responses as a single agent in the syngeneic tumor models established in human VISTA-Knockin (hVISTA-KI) mice. KVA12123 also remodeled the TME from an immunosuppressive to an antitumorigenic, proinflammatory phenotype by activating myeloid cells, leading to T and NK cell recruitment and activation. This mechanism of action drives a strong anti-tumor single-agent efficacy that can be further enhanced in combination with either anti-PD(L)1 or anti-CTLA-4 treatment. KVA12123 is currently being evaluated in a Phase 1/2 clinical trial as a monotherapy and in combination with pembrolizumab in patients with advanced solid tumors.

## 2 Materials and methods

### 2.1 Antibody library generation

Fully human scFv antibodies directed against human VISTA were generated after immunization of humanized Trianni® mice and sorting natively-linked B cell scFv repertoires. Briefly, transgenic humanized Trianni mice were immunized with soluble human VISTA-His extracellular domain (R&D Systems). B cells were isolated from spleen, lymph nodes, and bone marrow. B cells were then encapsulated into droplets with oligo-dT beads and a lysis solution, followed by overlap-extension RT-PCR to generate a DNA amplicon that encodes the scFv libraries with native pairing heavy and light Ig. The scFv libraries were then transfected into yeast cells for surface display, stained with biotinylated VISTA-His protein and Streptavidin-PE conjugate (Life Tech), and scFvs binding to VISTA were sorted by FACS (BD Influx). Finally, deep sequencing (Illumina) was used to identify all clones in the pre- and post-sort populations.

### 2.2 Production and characterization of monoclonal antibodies in ExpiCHO cells

Monoclonal antibodies were expressed in ExpiCHO-S cells (Thermo Fisher) cultured and transfected according to the manufacturer’s specifications. Antibodies were purified from culture supernatant using GE AKTA Pure FPLC and MabSelect PrismA Resin (Cytiva) using 20mM Acetate, 30mM Glycine pH 3.75 elution buffer. Purified antibodies were buffer exchanged with dialysis cassettes into PBS pH 7.4 buffer and evaluated by SEC-HPLC at 220 nm on a Tosoh TSKgel G3000SWXL column using 0.2 M sodium phosphate pH 6.7 as the mobile phase to determine monomeric purity. Endotoxin levels were measured using a kinetic chromogenic LAL assay with the Charles River Endosafe PTS100. The VSTB174 antibodies were derived from the Janssen Pharmaceuticals VSTB174 sequence (WO2016207717) and were expressed as full human IgG1 antibodies.

### 2.3 Kinetics of KVA mAbs binding to human, monkey, and mouse VISTA

The binding kinetics of KVA mAbs to VISTA was determined by capturing KVA mAbs with an anti-human Fc antibody immobilized on an AHC chip (Octet BLI platform, Sartorius). Briefly, 20 ug/mL of KVA mAbs in PBS were loaded onto an anti-human IgG Fc capture (AHC) biosensor for 120 seconds. The loaded biosensor was then incubated with 50nM of monomeric VISTA-ECD in PBS for a 240-second association period and transferred to PBS for a 360-second dissociation period. A 1:1 global curve fitting analysis was performed to determine equilibrium (KD), association (ka), and dissociation (kdis) rate constants. The binding kinetics of KVA12.2a (WT, mIgG2a) and hVISTA-ECD-Fc interactions were determined by capturing KVA12.2a with an anti-mouse IgG Fc capture (AMC) biosensor.

### 2.4 Enzyme−linked immunosorbent assays

Five different ELISAs were performed. In all cases, plates were incubated with streptavidin-HRP (R&D Systems) and developed with TMB colorimetric substrate (Thermo Scientific) according to the manufacturer’s instructions. Absorbance values at 450 nm were detected on a CLARIOstar plate reader (BMG). The values of half-maximal effective concentration (EC50) or inhibitory concentration (IC50) were calculated using nonlinear regression fitting with GraphPad Prism. (1) The nickel-coated plate ELISA with hVISTA-ECD-His tag and KVA mAbs: the binding of KVA mAbs to 50 ng/well of hVISTA-ECD-His tag was detected using nickel-coated plates (Thermo Scientific) pre-blocked with BSA (bovine serum albumin). The plate-captured hVISTA-ECD His tag (Sino Biologics) was incubated with KVA mAbs for one hour at RT, and then the plates were washed three times with PBS, 0.05% Tween 20. KVA mAbs were diluted with 0.5% BSA, 0.05% Tween 20, PBS buffer, and incubated for one hour at RT. Plates were washed and incubated with biotinylated anti-human IgG (H+L) (Jackson ImmunoResearch). (2) ELISA with KVA mAbs and the B7 family cell-surface proteins: MaxiSorp 96-well plates were coated with 2 ug/mL of human B7-1 (CD80), B7-2 (CD86), B7-H1 (PD-L1), B7-H2 (ICOS), B7-H3 (CD276), B7-H4 (VTCN1), B7-H6, B7-H6 or B7-H5 (VISTA) his-tag proteins (Sino Biological), blocked and incubated with 10 ug/mL of KVA mAbs or positive control antibodies for 1 hour at RT. Plates were washed and incubated with a biotinylated anti-human IgG (H+L) (Jackson ImmunoResearch). (3) The competitive ELISA to measure KVA mAb inhibition of VISTA binding to VSIG3, VSIG8, PSGL1, or LRIG1: KVA12123 or isotype control antibodies were incubated with h-VISTA-ECD-Fc-Avi-tag (R&D Systems) for one hour at room temperature and then transferred to MaxiSorp 96-well plates coated with human VSIG3, VSIG8, PSGL1, or LRIG1 proteins (R&D Systems) and incubated for two hours. pH 6.0 dilution and wash buffers were used for the PSGL1 ELISA and pH 7.4 for the VSIG3, VSIG8, and LRIG1 ELISAs. (4) ELISA with KVA mAbs and hVISTA-ECD at different pH: MaxiSorp 96-well ELISA plates (Thermo Scientific) coated with 50ng/well of hVISTA-ECD-His tag (Sino Biological) were blocked and incubated with KVA mAbs at pH 6.0, 6.5, 7.0, or 7.4. Bound KVA mAbs were detected using the biotinylated anti-human IgG (H+L) (Jackson ImmunoResearch). (5) ELISA with KVA mAbs and hVISTA-ECD-Fc mutants for epitope mapping: Maxisorb 96-well plates coated with hVISTA-ECD-Fc were blocked and incubated with KVA mAbs. Bound KVA mAbs were detected using the biotinylated anti-human IgG light chain (Jackson ImmunoResearch).

### 2.5 hVISTA-ECD-Fc mutagenesis and purification

DNA encoding human VISTA-ECD (33-194, Uniprot) was cloned into an Fc (human IgG1) construct (pcDNA3.3 vector) that contained a Factor Xa cleavage site at the N-terminus of the hinge region. The hVISTA-ECD-Fc mutants were generated using site-directed mutagenesis using a standard two-stage QuikChange PCR protocol. Human VISTA-ECD-Fc proteins were expressed in human Expi293F (Thermo Fisher) cultivated and transfected according to the manufacturer’s specifications. The hVISTA-ECD-Fc proteins were purified from culture supernatant using GE AKTA Pure FPLC and MabSelect PrismA Resin (Cytiva).

### 2.6 AlphaLISA Fc receptor competition binding assay

AlphaLISA Fc receptor binding kits (FCGR1 (CD64), #AL3081; FCGR2A (167H) (CD32a), #AL3086; FCGR2A (167R) (CD32a), #AL3087; FCGR3A (176Phe/F158) (CD16a), #AL347; FCGR3A (176Val/V158) (CD16a), #AL348; FcRn, #AL3095) were supplied by PerkinElmer. All assays were performed as instructed in the protocol in each kit’s technical data sheet, in white 96-well ½ area plates (PerkinElmer # 6005560). All FcGR binding assays were run in AlphaLISA HiBlock Buffer, and the FcRn binding assay was run in AlphaLISA MES Buffer (supplied in each kit). Plates were measured using the CLARIOstar plate reader (BMG). IC50 values were calculated by using nonlinear regression fitting with GraphPad Prism.

### 2.7 Flow cytometry-based cellular binding assay

KVA antibodies were evaluated for their ability to bind human, mouse, and cynomolgus monkey VISTA stably expressed on CHO-K1 cells (ATCC). VISTA-expressing CHO-K1 cells were established via the selection of stably transfected clones. Cells were cultured according to ATCC specifications, harvested, and incubated with 150, 1, or 0.05 nM KVA mAbs for 30 min on ice, followed by additional washes and staining with PE-conjugated goat anti-human IgG (Jackson ImmunoResearch) for 30 minutes on ice. Cells were fixed with Cytofix (BD Pharmingen) for 5 min at room temperature. Mean fluorescence intensity levels were determined using flow cytometry (Thermo Fisher Attune NxT).

### 2.8 MDSC-mediated T-cell suppression assay

Frozen primary human PBMCs (AllCells) were obtained from healthy donors. MDSCs were obtained using purified CD11b+ cells derived from human PBMCs after treatment with 10 ng/mL GM-CSF (Thermo Fisher) and 10 ng/mL IL-6 (Thermo Fisher) for 7 days. PBMCS were labeled with CellTrace™ Violet (CTV) according to the manufacturer’s protocol (Thermo Fisher, C34557). MDSCs were then co-cultured with CTV-labelled autologous PBMCs at a 1:1 ratio in the presence of 1 µg/mL anti-human CD3 antibody (OKT3, Biolegend), 100 µg/mL KVA12123 or isotype control for 96 hours at 37°C and 5% CO_2_. The absolute cell counts were determined by flow cytometry (Thermo Fisher Attune NxT), and cytokine secretion was determined using the culture supernatant (R&D Systems, DY285B and DY210). CTV profiles in the human CD45+ human CD3+ gate were analyzed.

### 2.9 Monocyte activation assay

CD14+ monocytes were incubated with autologous human PBMCs (AllCells) and 10 µg/mL KVA mAbs. After 24-hour incubation, cells were harvested and stained with the cell surface antibodies to evaluate the upregulation of HLA-DR and CD80 on CD14+ monocytes. CXCL-10 chemokine secretion was analyzed in the cell supernatants (R&D Systems, DY266).

### 2.10 SEB-mediated T-cell activation assay

Human PBMCs (AllCells) were depleted of NK cells (Miltenyi Biotec) and cultured with 5 ng/mL staphylococcal enterotoxin B (SEB) superantigen in the presence of 3 – 0.03ug/mL of KVA12123 or an isotype control antibody in X-VIVO™-15 medium (Lonza). After 4 days of incubation at 37°C, the supernatants were collected to measure IFNγ (R&D Systems).

### 2.11 Pharmacokinetics studies in hVISTA-KI mice

Sixteen- to twenty-week-old male or female hVISTA-KI (genOway, Lyon, France) mice received a single 10mg/kg, 30mg/kg, or 100mg/kg i.p. injection of KVA mAbs, and blood sampling was performed at 2, 4, 8, 12, 24, 48, or 72-hour time points (n=2 mice per time point). Mice were anesthetized with isoflurane anesthesia, and whole blood was collected by retro-orbital, submental, or terminal cardiac puncture methods into serum separator tubes (BD Microtainer). Blood was allowed to clot at RT for 30-60 minutes and then centrifuged (10,000 x g for 7 minutes). The serum was aliquoted into individual tubes and then frozen at -80°C until analysis. Concentrations of KVA mAbs in serum were determined by ELISA. MaxiSorp 96-well ELISA plates (Thermo Scientific) were coated with 50 ng/well of VISTA-ECD-His tag (Sino Biological) overnight at 4°C, blocked, and treated with serum samples diluted 1:10, 1:100, 1:1,000, 1:10,000, or 1:100,000. Standard curves (0.5-1000ng/mL) for each KVA mAbs were prepared using 0.1%, 1%, and 10% mouse serum. Bound KVA mAbs were detected using the biotinylated anti-human IgG (H+L) (Jackson ImmunoResearch) and with streptavidin-HRP (R&D Systems) and developed with TMB colorimetric substrate (Thermo Scientific) according to the manufacturer’s instructions. Absorbance values at 450 nm were detected on a CLARIOstar plate reader (BMG). GraphPad Prism was used to determine the concentration value for each sample using nonlinear regression fitting with a sigmoidal dose-response (variable slope). Pharmacokinetic parameters were calculated using non-compartmental analysis after extravascular input using PKSolver 2.0 software.

### 2.12 Syngeneic tumor studies

Human VISTA knock-in mice (hVISTA-KI, genOway, Lyon, France) were generated and validated as described by Johnston et al., 2019 (18). MB49 (Millipore-Sigma), MC38 (National Institutes of Health), E.G7-OVA (ATCC), CT26 (ATCC), and B16-F10 (ATCC) cell lines were cultured according to the vendor’s guidelines. Eight- to twenty-week-old female hVISTA-KI mice were inoculated subcutaneously in the right flank with tumor cells (5x10^5^ cells for MB49 cells, 1x10^6^ cells for E.G7-OVA). Eight- to ten-week-old female BALB/cJ mice (The Jackson Laboratory) were inoculated with 5x10^5^ CT26 cells subcutaneously in the right flank with tumor cells. Eight- to ten-week-old female C57Bl/6 mice (The Jackson Laboratory) or hVISTA-KI mice were inoculated subcutaneously in the right flank with tumor cells (3x10^5^ cells for B16-F10 cells, 5x10^5^ cells for MC38 cells). In all cases, after reaching an average volume of 75 mm3, tumors were measured, and mice were randomized by tumor volumes into groups into treatment groups. In all experiments, treatments were administered intraperitoneally (i.p.). In the MC38 model, hVISTA-KI mice were dosed with 10 mg/kg of KVA12.2 (WT, mIgG2a) or isotype control (mIgG2a) 3x/week as a monotherapy or in combination with 5 mg/kg anti-mPD-1 (Bio X Cell, Clone RMP1-14) 2x/week for three weeks. In the MB49 and E.G7-OVA models, hVISTA-KI mice were dosed with 3-30 mg/kg anti-VISTA antibody or isotype control (hIgG1, hIgG4 or mIgG2a) or in combination with 5 mg/kg anti-mPD-1 (Bio X Cell, Clone RMP1-14) 2x/week for 3 weeks. In the B16-F10 model, C57Bl/6 mice were dosed with 15 mg/kg anti-mVISTA (Bio X Cell, Clone 13F3) or isotype control and/or 10 mg/kg of anti-mPD-L1 3x/week. In the CT26 model, BALB/cJ mice were dosed with 15 mg/kg anti-mVISTA (Bio X Cell, Clone 13F3) or isotype control and/or 10 mg/kg of anti-mPD-L1 (Bio X Cell, Clone 10F.9G2) and/or 10 mg/kg of anti-mCTLA-4 (Bio X Cell, Clone 9D9) 3x/week. For all experiments, tumor sizes were measured 3x/week using digital calipers. Tumor volume was calculated using the formula (Y × X × X)/2, where Y is the longest dimension, and X is the perpendicular dimension. The study procedures were carried out in accordance with the Guide for the Care and Use of Laboratory Animals of the National Institutes of Health. The protocols were approved by the Kineta Inc. and Crown Bioscience Institutional Animal Care and Use Committee (IACUC). Mice were continuously monitored for symptoms of illness with changes to posture, activity, breathing, and fur texture and euthanized when clinical symptoms reached the cumulative limit outlined by animal ethics.

### 2.13 *Ex-vivo* analysis of tumor microenvironment

Tumor samples were harvested from hVISTA-KI mice treated with hIgG1 isotype control or KVA12123 on day 12 (24 hours after the third dose). Tumor tissues were dissociated into single-cell suspension using the tumor dissociation kit (Miltenyi Biotec) according to the kit’s specifications. Cells were then blocked with mouse Fc block (BD Biosciences), stained with the fixable live/dead near-IR dye (Thermo Fisher), anti-mouse myeloid and lymphoid markers (Biolegend), and fixed using BD Cytofix™ Fixation Buffer (BD Biosciences). The following markers were used for the TAM/MDSC panel: CD11b (M1/70), Ly6C (HK1.4), F4/80 (BM8), CD163 (S15049l), Ly6G (1A8), CD80 (16-10A1), CD45 (30-F11), I-A/I-E (M5/114.15.2), and EPCAM (G8.8). The following markers were used for the DC panel: CD14 (Sa14-2), F4/80 (BM8), CD11c (N418), I-A/I-E (M5/114.15.2), XCR1 (ZET), CD103 (2E7), CD11b (M1/70), CD8 (53-6.7), EPCAM (G8.8), and CD45 (30-F11). The following markers were used for the lymphoid panel: CD3 (1452C11), CD4 (GK1.5), CD8 (53-5.8), NK1.1 (PK136), CD44 (IM7), CD62L (MEL-14), TIM-3 (RMT3-23), CD69 (H1.2F3), EPCAM (G8.8), and CD45 (30-F11). Cells were analyzed via flow cytometry (Thermo Fisher Attune NxT). Data analysis was performed using FlowJo 10.7.1 software.

### 2.14 Pharmacokinetics in cynomolgus monkeys

Protein naïve female cynomolgus monkeys received a single 30mg/kg or 100mg/kg intravenous (i.v.) administration of KVA12.1 or KVA12123. Blood sampling was performed before dose administration and then 0.083, 1, 6, 12, 24, 72, 96, 144, 168, 216, 264, 336, and 672 hours post-dose (n=1 monkey per time point). Blood was collected from the femoral artery or vein into serum separator tubes. Blood was allowed to clot at room temperature before centrifugation, and then serum was aliquoted into individual tubes and frozen at -60°C to -90°C until analysis. Concentrations of KVA mAbs in serum were determined by ELISA using the same method as was used for mouse PK studies, except cynomolgus monkey serum was used in the standard sample preparation. The studies were conducted at Charles River Laboratories (Mattawan, MI). The studies were approved by the Charles River Laboratories Institutional Animal Care and Use Committee (IACUC) and were conducted in accordance with the Animal Welfare Act and the National Institute of Health guidelines.

### 2.15 Toxicology studies in cynomolgus monkeys

A 4-week repeat dose study with a 4-week recovery period was performed to assess the potential toxicity of KVA12123 in cynomolgus monkeys. Naïve 1.8-5 kg male and female cynomolgus monkeys (n=3-5 per sex per group) received an i.v. bolus injection of KVA12123 every 7 days for 4 doses at 10mg/kg, 30mg/kg, or 100mg/kg. The following parameters and endpoints were evaluated in this study: clinical observations, body weights, qualitative food consumption, injection site observations, ophthalmology, veterinary physical examinations, jacketed external telemetry (JET), respiratory rates (visual), body temperature, neurological examination, blood pressure, clinical pathology parameters (hematology, coagulation, clinical chemistry, and urinalysis), bioanalytical and toxicokinetic parameters, anti-drug antibody, cytokine analysis, immunophenotyping, organ weights, and macroscopic and microscopic examinations. The studies were approved by the Charles River Laboratories Institutional Animal Care and Use Committee (IACUC) and were conducted in accordance with the Animal Welfare Act and the National Institute of Health guidelines.

### 2.16 Antibody-dependent cell cytotoxicity assay

A dose-titration was performed to test KVA antibodies (100 pg/mL - 10 mg/mL) in the ADCC assay with Effector cells (PBMCs from healthy donors treated with 200 U/mL of IL-2 O/N) and Target cells (hVISTA-Raji cells) with a [12:1] ratio. Cell death was detected using CytoToxGlo™ reagent (Promega) after a 4-hour incubation. Relative luminescent units (RLU) were measured using a ClarioStar Plus plate reader (BMG Labtech). A fold increase of dead cells over untreated negative control was plotted.

### 2.17 Complement-dependent cytotoxicity assay

Raji or hVISTA-Raji cells were seeded at 100,000 cells/well onto a white 96-well flat bottom tissue culture assay plate in 100 µL of X-Vivo-15 medium (Lonza). Antibodies were serially diluted in the X-Vivo-15 medium in a 96-well V-bottom polypropylene plate. 50 µL of antibodies were transferred into assay plate wells. 50 µL of human universal AB serum (Sigma) was added to the assay plate wells. The assay plates were then incubated for 6 hours in the 37^0^C, 5% CO_2_ humidified incubator. The cells were assayed using a CellTox-Glo Cytotoxicity Assay Kit (Promega), and the data were read using ClarioStar Plus (BMG Labtech). Rituximab was used as a positive control. A fold increase of dead cells over untreated negative control was plotted.

### 2.18 Whole blood cytokine release assay

Fresh heparinized whole blood (175 μl/well) from eleven healthy human donors was added to the 96-well polystyrene round-bottom sterile plates (Corning)and incubated with 25 μl of 1 to 1000 µg/ml KVA mAbs in X-Vivo-15 medium (Lonza) for 24 hours at the 37°C, 5% CO2 humidified incubator. Plates were centrifuged at 1,100 g for 5 minutes, and blood plasma supernatants were collected and transferred to 96-well polypropylene V-bottom plates and stored at −20°C. Cytokine levels in plasma supernatants were detected using a Milliplex human cytokine magnetic bead panel following manufacturer instructions. Duplicate plasma supernatants derived from the whole blood assay were analyzed using Luminex. Human hIgG1 isotype and Cetuximab (Bio X Cell SIM0002) antibodies were negative controls. Positive controls included anti-CD28 ANC28.1/5D10 (EMD Millipore), Alemtuzumab (Ichorbio ICH4002), and anti-human CD3/CD28 (Stem Cell Technologies) antibodies.

### 2.19 Statistical analysis

All graphs and binding curve regressions were created using GraphPad Prism software. The number of replicates is specified in the figure legends for all studies. Error bars represent the standard deviation (SD) from the mean or standard error of the mean (SEM) as specified in the figure legends. P values were calculated by unpaired t-test. *P < 0.05, **P < 0.01 and ***P < 0.001 are considered statistically significant.

## 3 Results

### 3.1 Highly diverse fully human anti-VISTA mAbs show specific binding to human and cynomolgus monkey VISTA with similar potency

**Figure 1:**
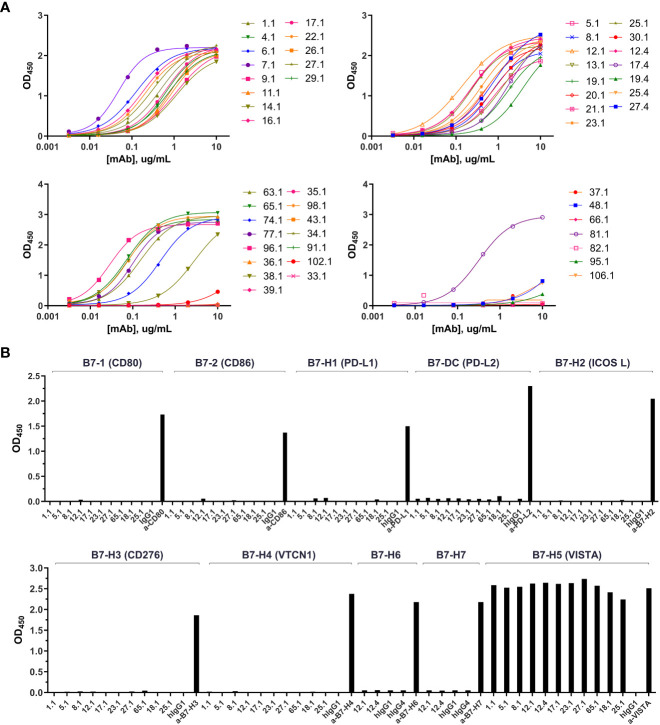
KVA mAbs bind VISTA with high affinity and specificity. **(A)** The binding of KVA mAbs to plate-coated hVISTA-ECD (n=1), evaluated by ELISA. **(B)** The binding of KVA mAbs to the B7 family cell-surface proteins, tested by ELISA. Data are shown as means ± SD (n=2). KVA mAbs on a human IgG1 or IgG4 backbone are indicated by.1 or.4, respectively.

Fully human ScFv antibodies directed against human VISTA were generated after immunization of humanized Trianni® mice with soluble human VISTA-ECD. One hundred and seven natively-paired fully human single-chain variable fragments (scFv) directed against human VISTA were generated with high diversity in both heavy and light chains. Pairwise alignments were performed using Clustal Omega (19) to cluster scFv sequences into clades based on similarity in the CDR3 regions (**Figure S1**). CDR3 alignments were used because they exhibit the greatest diversity compared to CDR1 and CDR2 regions. Fully reconstructed human IgG1 anti-VISTA monoclonal antibodies (KVA mAbs), representative of all clades, were produced in ExpiCHO cells to evaluate their binding characteristics. Most of the tested antibodies demonstrated potent binding to hVISTA-ECD by ELISA with low nanomolar half-maximum effective concentration (EC50) (**Figure 1A** and **Table S1**) as well as a fast Ka (association) and a slow Kdis (dissociation) for hVISTA-ECD determined by bio-layer interferometry (BLI) (**Tables S1** and **S2**). We also evaluated the cross-reactivity of KVA mAbs to cynomolgus monkey and mouse VISTA-ECD. Almost every KVA antibody bound equivalently to human and cynomolgus VISTA, and none demonstrated significant binding to mouse VISTA (**Tables S1** and **S2**). These results were confirmed by flow cytometry on CHO-K1 cell lines transfected with either human, cynomolgus monkey or mouse VISTA, where KVA mAbs recognized the cell surface-expressed human and cynomolgus VISTA but not mouse VISTA (**Figure S3**). To further characterize the specificity of KVA antibodies, we evaluated their ability to bind to the related members of the B7 protein family by ELISA: CD80/B7-1, CD86/B7-2, ICOS/B7-H2, PD-L1/B7-H1, B7-DC/PD-L2/CD273, B7-H3/CD276, B7-H4/B7S1/B7x, B7-H6, and B7-H7. KVA mAbs did not bind to any members of the B7 family of cell surface receptors other than VISTA (**Figure 1B**). This data demonstrates the strong affinity and specificity of most of the tested KVA mAbs.

### 3.2 KVA12.1, anti-VISTA IgG1 mAb, exhibits an extended serum half-life relative to other anti-VISTA mAbs

**Figure 2:**
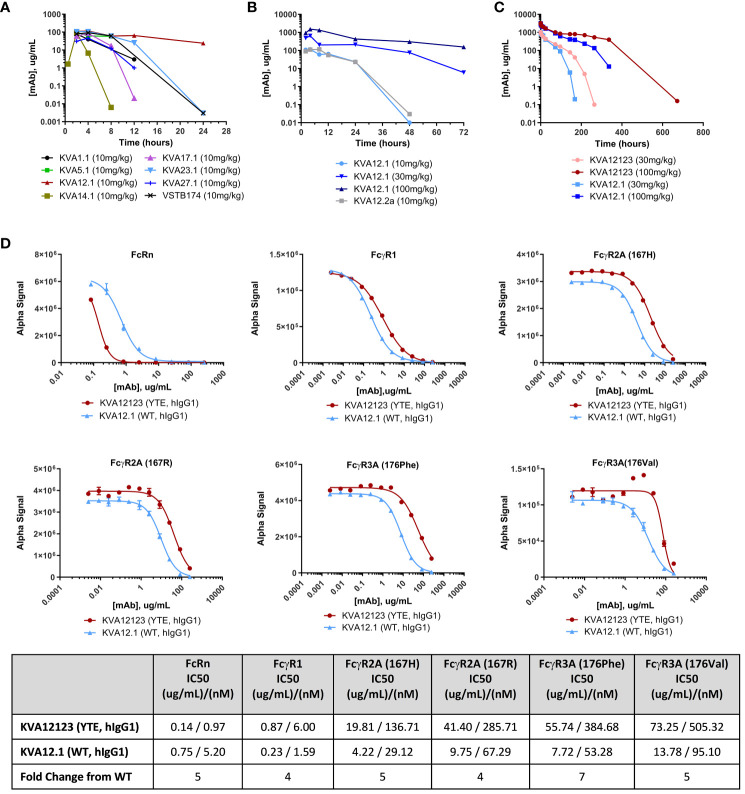
KVA12.1 (WT, hIgG1) and KVA12.2a (WT, mIgG2a) show extended half-lives in VISTA-KI mice, and KVA12123 (YTE, hIgG1) demonstrates longer serum half-life compared with WT KVA12.1 in cynomolgus monkeys due to improved recycling via interaction with FcRn. **(A)** A representative pharmacokinetic profile of KVA mAbs in plasma of hVISTA-KI mice following a single 10 mg/kg i.p. infusion. Data are shown as means ± SD (n=2). **(B)** PK parameters of KVA12.1 (WT, hIgG1) and KVA12.2a (WT, mIgG2a) mAbs in plasma of hVISTA-KI mice following a single i.p. dose at 10 mg/kg, 30 mg/kg or 100 mg/kg. **(C)** PK parameters of KVA12123 (YTE, hIgG1) and KVA12.1 (WT, hIgG1) mAbs in serum of female cynomolgus monkeys following a single i.v. dose. Serum was sampled at pre-dose and 0.083, 1, 6, 12, 24, 72, 96, 144, 168, 216, 264, 336, and 672-hour post-dose time points (n=1). Saturation of target-mediated metabolism is evident at higher doses. **(D)** KVA12123 (YTE, hIgG1) and KVA12.1 (WT, hIgG1) competitive binding to FcRn (at pH 6.0), FcgR1, FcgR2A (167H or 176R) or FcgR3A (176Phe or 176Val) (at pH 7.4). IC50s were calculated by using four-parameter nonlinear regression. Data are shown as means ± SD (n=2).

The pharmacokinetic (PK) profile of KVA mAbs was evaluated in hVISTA-KI mice to identify a lead candidate with the least clearance relative to other KVA antibodies. The intent was to minimize target-mediated drug metabolism (TMDD) associated with the relatively high expression of VISTA in the central compartment. Male or female hVISTA-KI mice were administered a single 10 mg/kg intraperitoneal (i.p.) injection of anti-VISTA antibodies. Serum samples were analyzed by ELISA to determine anti-VISTA antibody concentrations over time following i.p. administration (**Figure 2A**), and single-dose PK parameters were determined (**Table S3**). For all tested antibodies, serum concentrations peaked between 2 and 4-hours post-dose with peak serum concentrations (C_max_) ranging from 46-115 ug/mL. KVA12.1 (wild type (WT), hIgG1) showed the highest C_max_ and the longest serum half-life relative to other tested anti-VISTA antibodies. We compared KVA mAbs with VSTB174 (Janssen Therapeutics), the first anti-VISTA mAb used in human clinical trials (20). VSTB174 exhibited a 7-fold shorter half-life than KVA12.1.

We then investigated the PK profile of KVA12.1 (WT, hIgG1) after administering increasing doses of the antibody in hVISTA-KI mice. KVA12.1, administered at 30 mg/kg, resulted in 645 ug/mL Cmax, a 6-fold increase over the 10 mg/kg dose (**Figure 2B** and **Table S3**). The KVA12.1 AUC0-t at 30 mg/kg increased 8-fold over the 10 mg/kg dose. Administration of KVA12.1 at 100 mg/kg demonstrated a 2-fold increase of Cmax, a 3-fold increase of AUC0-t, and a 3-fold increase of half-life over the 30 mg/kg. The nonlinear pharmacokinetics of KVA12.1 mAbs is consistent with target-mediated drug disposition (TMDD) (21). TMDD accounted for a significant portion of KVA12.1 mAb clearance at lower concentrations. However, target saturation was observed at higher doses.

PK parameters of KVA12.2a (WT, mIgG2a) were similar to KVA12.1 (WT, hIgG1) (**Figure 2B** and **Table S3**). Overall, KVA12.1 (and the mouse surrogate KVA12.2a) showed the best pharmacokinetic profile of all the screened antibodies with the highest peak serum concentration (C_max_), the highest total exposure (AUC0-t), and the longest half-life. Based on the results obtained from the *in vitro* screen and the *in vivo* PK evaluation, KVA12.1 was selected as the lead anti-VISTA antibody.

### 3.3 KVA12123, the YTE variant of KVA12.1, demonstrates extended serum half-life in cynomolgus monkeys

FcRn-mediated recycling is a critical factor that can influence a monoclonal antibody’s pharmacokinetics (21). To further improve the half-life of KVA12.1 mAb, a YTE triple mutation (M252Y, S254T, T256E) was introduced in the Fc portion of KVA12.1 to increase binding to FcRn. The YTE mutation has been shown to increase the binding of antibodies by 10-fold to cynomolgus and human FcRn at pH 6.0 and as a correlate to increase the serum half-life by 4-fold in cynomolgus monkeys (22–25). FcRn prevents IgG degradation by efficiently sorting bound IgG into recycling endosomes and away from lysosomes. Another advantage of YTE mutation is the potential reduction of antibody-dependent cell-mediated cytotoxicity (ADCC), possibly leading to a reduced therapeutic antibody side effect profile (22).

The PK and tolerability of KVA12123 (YTE, hIgG1) and KVA12.1 (WT, hIgG1) were evaluated in cynomolgus monkeys with a 28-day observation period. Female cynomolgus monkeys were administered a single dose of 30 mg/kg or 100 mg/kg IV injection of KVA12.1 or KVA12123, and blood serum sampling was performed at different time points (**Figure 2C** and **Table S4**). Both KVA12.1 and KVA12123 demonstrated dose-proportional increases in Cmax and greater than dose-proportional increases in AUC0-t. The initial (alpha) and beta half-life values were also extended for both antibodies at the 100 mg/kg dose compared to the 30 mg/kg dose due to saturation of the receptor-mediated metabolism at the higher doses. The initial half-life for KVA12.1 and KVA12123 was 30 and 48 hours at 30 mg/kg and 103 and 165 hours at 100 mg/kg, respectively. Our results demonstrated the reduced clearance and extended half-life of KVA12123 (YTE, hIgG1) relative to KVA12.1 (WT, hIgG1), potentially due to an enhanced FcRn-dependent recycling of KVA12123 mAbs (22). To confirm, the binding affinity of KVA12.1 and KVA12123 to FcRn was tested *in vitro*. KVA12123 demonstrated >5-fold stronger binding to the FcRn at pH 6.0 than KVA12.1 using AlphaLISA (**Figure 2D**). This was confirmed by results generated using BLI where KVA12123 showed a 9-fold higher affinity for FcRn at pH 6.0 than KVA12.1 (**Table S5**). Additionally, KVA12123 binding to FcγRI, FcγRIIa, and FcγRIIIa was significantly reduced when compared to KVA12.1 (WT, hIgG1). The reduced binding of KVA12123 to proinflammatory Fc receptors has significant functional effects *in vivo* for ensuring our clinical candidate’s low or no adverse immunoreactivity, as demonstrated later in our preclinical toxicology studies.

### 3.4 KVA12123 selectively inhibits the interaction of VISTA with its binding partners LRIG1, VSIG3, VSIG8, and PSGL1 and binds strongly to VISTA at neutral and acidic pH

**Figure 3:**
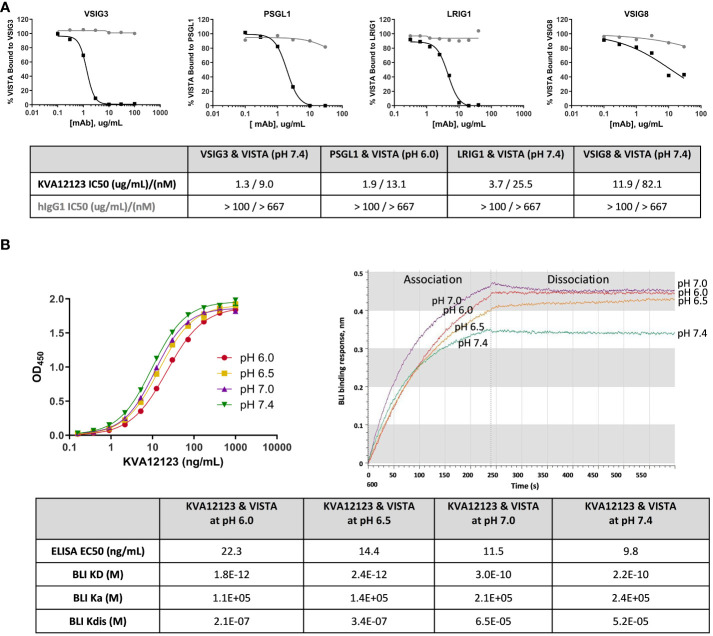
KVA12123 inhibits VISTA binding to VSIG3, VSIG8, PSGL1, and LRIG1 and binds strongly to VISTA at neutral and acidic pH. **(A)** Inhibition of VISTA interactions with its binding partners by KVA12123 (black squares) or hIgG1 (grey circles), evaluated by a competition ELISA. **(B)** The binding of soluble KVA12123 to plate-coated hVISTA-ECD-Fc at different pH, tested by ELISA (left). EC50s were calculated by using a four-parameter nonlinear regression fitting. Data are shown as means ± SD (n=2). The BLI binding sensorgrams for KVA12123 and monomeric hVISTA-ECD interactions at different pH (right). A 1:1 global curve fitting analysis was performed to determine equilibrium (KD), association (ka), and dissociation (kdis) rate constants. These data are representative of three independent experiments.

VSIG3, VSIG8, PSGL1, and LRIG1 were previously described as binding partners for VISTA (18, 26–28). VSIG3, VSIG8, and LRIG1 bind to VISTA at neutral pH, while PSGL-1 binds only at acidic pH (**Figure S4**). KVA12123 was evaluated in a competition ELISA to test its ability to inhibit the binding of VISTA to each of these proteins at pH 7.4 or pH 6.0 for PSGL1. KVA12123 effectively prevented the binding of VISTA to VSIG3, PSGL1, LRIG1, and VSIG8 with IC50 values of 9 nM, 13 nM, 26 nM, and 82nM respectively (**Figure 3A**). The pH in the tumor microenvironment can vary from neutral pH 7.4 to more acidic pH 5.5, depending on the level of hypoxia and glycolysis (29–31). Therefore, we evaluated KVA12123 binding to VISTA at neutral and acidic pH using both ELISA and BLI approaches. KVA12123 showed strong and similar binding to VISTA at all tested pHs from pH 6.0 to pH 7.4 (**Figure 3B**). This demonstrates that KVA12123 can interact with VISTA, preventing binding to its respective ligands at neutral and acidic pH.

### 3.5 KVA12123 binds to a unique epitope on VISTA

**Figure 4:**
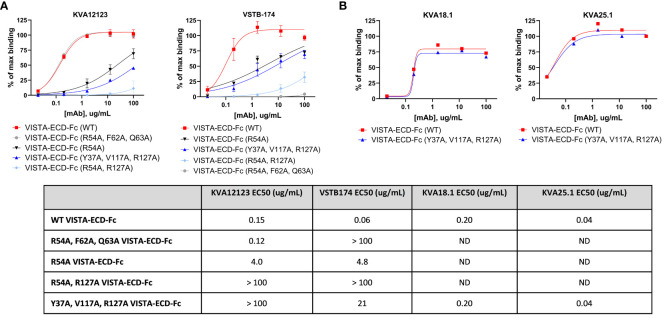
KVA12123 mAbs bind to a unique epitope on VISTA (Y37, R54, V117, and R127). **(A)** The binding of soluble KVA12123 or VSTB174 to plate-coated WT or mutant hVISTA-ECD-Fc proteins, analyzed by ELISA and expressed as a percentage of positive control (% of max binding). **(B)** The binding of soluble KVA18.1 or KVA25.1 mAbs from a different epitope bin to plate-coated WT or mutant hVISTA-ECD-Fc proteins, analyzed by ELISA and. EC50s were calculated by using four-parameter nonlinear regression. Data are shown as means ± SD (n=3). ND stands for not determined. These data are representative of three independent experiments.

We investigated the binding epitope of KVA12123 using mutation analysis of surface-exposed amino acid residues of human hVISTA-ECD. We generated a panel of solubly-expressed hVISTA-ECD-Fc mutants using alanine substitution (**Table S6** and **S7**) and evaluated the effect of these substitutions on KVA12 binding by ELISA and BLI approaches. We found that the following four hVISTA-ECD mutations, Y37A, R54A, V117A, and R127A, reduced the affinity and binding of KVA12123 to human VISTA and constituted its unique epitope. The combined triple mutation Y37A, V117A, and R127A strongly reduced the binding of KVA12123 to hVISTA-ECD (**Figure 4A** and **Tables S6** and **S7**). To confirm the structural integrity of the triple mutant, we used KVA18.1 and KVA25.1, anti-VISTA antibodies that bind to a different VISTA epitope (**Figure 4B**). KVA18.1 and KVA25.1 binding was retained on the triple mutant (Y37A, V117A, R127A) with no significant difference compared to the WT hVISTA-ECD. Therefore, while Y37A, V117A, and R127A VISTA mutations abrogated binding to KVA12123, this triple mutation did not alter the VISTA 3D structure. Additional mutagenesis revealed that a single R54A mutation resulted in a significant binding reduction of KVA12123 to hVISTA-ECD, and the double R54A and R127A mutation completely abolished KVA121123 binding to hVISTA-ECD. The amino acid residues R54, F62, and Q63 are important for interacting with another anti-VISTA antibody, VSTB174, developed by another company (6). However, KVA12123 binding to VISTA was not affected by the single mutations F62A and Q63A, demonstrating that the KVA12123 epitope is shifted toward the R54-containing C–C’ loop and the R127-containing beta-strand on VISTA (**Figure S5**). This region encompasses the binding site for VSIG3 and possibly LRIG1 (32). We also showed that the binding of KVA12123 to VISTA was not affected by H121A, H122A, and H123A mutations (**Tables S6** and **S7**). This histidine-rich cluster along the rim of the VISTA extracellular domain is unique to this protein and is not found in the other B7 family members (7). This region mediates binding to PSGL-1 via charged interactions between PSGL-1 sulfated tyrosine and VISTA protonated histidine residues at acidic pH (18). KVA12123 does not bind to these histidines but interacts strongly with R127 and slightly with E125 (**Table S7**). These two amino acids are very close to the putative binding site of PSGL1 and probably explain why KVA12123 can also prevent the interaction of PSGL1 with VISTA. Lastly, we checked if VISTA glycosylation impacts KVA12123 binding. VISTA has five potential sites for N-glycan modification via an NXT/S motif (3). We found that none of the five mutations (N17Q, N59Q, N76Q, N96Q, N158Q) affected KVA12123 binding to hVISTA-ECD (**Tables S6** and **S7**). In conclusion, the KVA12123 epitope includes the two main amino acids, R54 and R127, supplemented by two more amino acids, Y37 and V117, which stabilize the interaction.

### 3.6 KVA12123 blocks VISTA expressed on MDSCs and reversed VISTA-mediated suppression of activated T-cells

**Figure 5:**
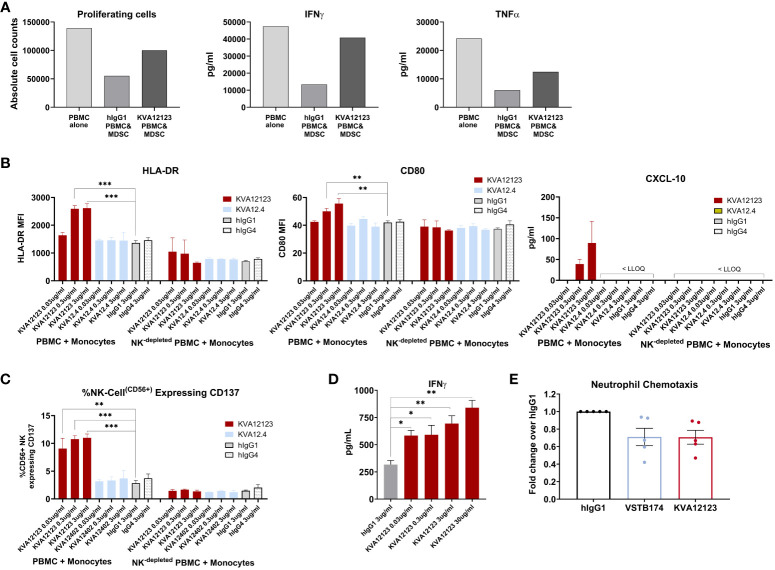
KVA12123 binding to VISTA on immune cells reverses MDSC immunosuppression, induces monocyte and NK cell activation, and prevents neutrophil chemotaxis. **(A)** KVA12123 reduces MDSC-mediated T cell suppression. MDSCs were obtained using purified CD11b+ cells derived from healthy donors PBMC after treatment with 10 ng/mL GM-CSF and 10 ng/mL IL-6 for 7 days. Cells were then co-cultured with CTVF-labelled autologous PBMCs and incubated with anti-CD3 antibody, 100 ug/mL KVA12123, or isotype control for 96 hours. These data are representative of two independent experiments. **(B)** Upregulation of HLA-DR, CD80, and CXCL-10 secretion by monocytes after treatment with 0.03, 0.3, and 3 ug/ml of KVA12123 (YTE, hIgG1), KVA12.4 (WT, hIgG4) or isotype controls (n=3). LLOQ is the lowest limit of quantitation. These data are representative of three independent donors. **(C)** Upregulation of CD137-activated NK cells in monocyte activation assay after treatment with 0.03, 0.3, and 3 ug/ml of KVA12123, KVA12.4, or isotype controls (n=3). **(D)** KVA12123 enhances SEB-mediated T-cell activation. Human NK-depleted PBMCs were induced with 5 ng/mL SEB and cultured in the presence of KVA12123 or isotype control for 4 days at 37°C, and the supernatant was analyzed for IFNγ secretion (n=3). **(E)** Inhibition of neutrophil migration by anti-VISTA antibodies. Human neutrophils were isolated from five healthy donors and incubated in the upper compartment of the chemotaxis chamber in the presence of KVA12123, VSTB174 antibodies, or isotype control at 1ug/ml. 50 ng/ml C5a was added to the lower chamber to evaluate the neutrophil chemotaxis activity. Data are shown as means ± SEM (n=5). P-value was obtained by unpaired t-test. *P < 0.05, **P < 0.01, ***P < 0.001.

We demonstrated that VISTA is highly expressed on myeloid cells, especially dendritic cells, monocytes/macrophages, and MDSCs (**Figure S6**). It has been previously shown in mouse tumor models that VISTA blocking decreases the migration of MDSCs into the TME (10) and possibly reduces MDSC-mediated suppression. We evaluated the effect of KVA12123 on MDSCs in a T cell suppression assay. Monocytes were differentiated into MDSCs for seven days using GM-CSF and IL-6 and then co-cultured with autologous PBMCs. An anti-CD3 antibody was added to the PBMC fraction to activate T-cells along with an isotype control antibody or KVA12123 (**Figure 5A**). Cells treated with KVA12123 showed restoration of T-cell proliferation associated with increased IFNγ and TNFα secretion after 96 hours compared to isotype control. This demonstrates that blocking VISTA on MDSCs with KVA12123 reverses T cell suppression and modulates the immunosuppression mediated by this cell population. Since MDSCs are one of the main drivers of immunosuppression in the TME, the KVA12123 blockade of VISTA on this cell population should help to restore an effective anti-tumor immune response.

### 3.7 KVA12123 induces monocyte and NK cell activation in an Fc-dependent manner

VISTA is predominantly expressed within the hematopoietic compartment, with the highest expression detected on myeloid lineage cells (11). To evaluate the effect of KVA12123 on the myeloid population, we have performed *in vitro* assays on CD14+ monocytes expressing high levels of VISTA on their surface (**Figure S6**). CD14+ cells were enriched from PBMCs and co-cultured with autologous PBMCs in the presence of KVA12123 or isotype control. KVA12123 induced a dose-dependent upregulation of HLA-DR and to a lower extent of CD80 on CD14+ cells at 0.03, 0.3, and 3 ug/mL compared to isotype control. This was also associated with an increase of the IFNγ-dependent cytokine CXCL-10 (**Figure 5B**). No induction was observed with KVA12.4, the human IgG4 version of KVA12. This indicates that a functional IgG1 Fc domain is necessary to illicit monocyte activation and differentiation of myeloid cells with an “antigen presentation cell” phenotype. This upregulation of activation markers on the surface of monocytes was completely lost in NK-depleted PBMCs, further indicating that NK cells that mediate Fc-binding and cross-linking are crucial for KVA12123 monocyte activation. Moreover, these NK cells exhibit a significant increase of the activation marker CD137 on their surface after treatment with KVA12123, while this was not observed when NK cells were incubated with KVA12.4 (WT, hIgG4) (**Figure 5C**). These data indicate that NK cells play an essential role in the mechanism of action of KVA12123.

### 3.8 KVA12123 increases IFNγ secretion in T-cells activated with a superantigen

To evaluate the functional effect of KVA12123 on T cells, a T-cell activation assay using Staphylococcus Enterotoxin B (SEB) superantigen was developed. PBMCs were incubated with a suboptimal dose of SEB, which directly links MHC class II protein on the surface of antigen-presenting cells (APC) to the T cell receptor (TCR), causing T cell activation and, subsequently, IFNγ secretion. Since IFNγ can also be secreted by NK cells, NK-depleted PBMCs were used in the assay to reduce the background signal and improve the dynamic range of the response. A dose-dependent increase of IFNγ secretion was observed in SEB-treated NK-depleted PBMCs when incubated with KVA12123 mAb for four days (**Figure 5D**). This implies that KVA12123 antagonistic binding to VISTA led to the potentiation of SEB-mediated T cell activation. This mechanism of action is complementary to KVA12123 function on myeloid cells and demonstrates the polyfunctional mechanism of our antibody on innate and adaptive immune cells.

### 3.9 KVA12123 inhibits neutrophil chemotaxis

It has been previously reported that VISTA blockade could dramatically impact chemotaxis of myeloid cells, preventing their migration (10). Neutrophils are known for their pro- and anti-tumor activities. A subpopulation of neutrophils, PMN-MDSCs, phenotypically similar to classical neutrophils, localize predominantly in the TME and exhibit immune-suppressive proprieties. We utilized a neutrophil chemotaxis assay to evaluate the effect of KVA12123 blockade on myeloid cell migration (**Figure 5E**). We assessed the anti-VISTA antibody VSTB174 for comparison, as it has been previously demonstrated to inhibit neutrophil migration (32). We observed that KVA12123 and VSTB174 anti-VISTA blocking antibodies reduced neutrophil chemotaxis by 25%, potentially leading to reduced migration of immune-suppressive MDSCs in the TME.

### 3.10 KVA12123 demonstrates a strong antitumor effect in VISTA-humanized mouse models as a single agent or in combination with other checkpoint inhibitors

Before evaluating the anti-tumor activity of our lead anti-VISTA antibody *in vivo* in hVISTA-KI mice, we conducted proof-of-concept experiments with an anti-mouse anti-VISTA antibody, clone 13F3, which has been previously shown to inhibit tumor growth (11). B16-F10 (melanoma) and CT26 (colon carcinoma) tumor models were evaluated in C57Bl/6 and Balb/c mice, respectively. These experiments aimed to assess combinations of an anti-VISTA antibody with different checkpoint inhibitors. We observed that anti-VISTA 13F3 antibody induced potent tumor growth inhibition (TGI) when used in combination with anti-mPD-L1 (B16-F10 model) or anti-mPD-L1 and CTLA-4 (CT26 model) (**Figure S7**).

**Figure 6:**
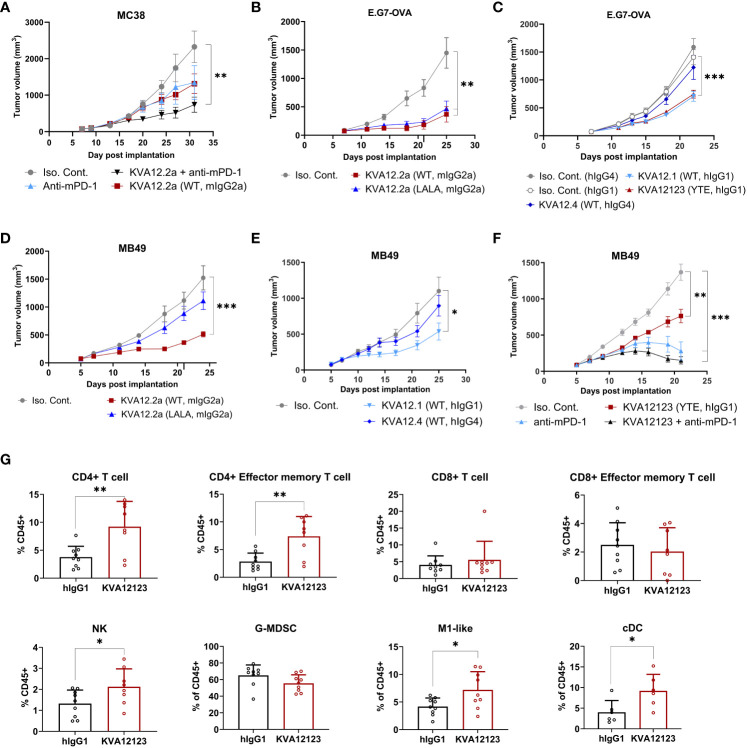
KVA12 demonstrates strong antitumor responses as a single agent or in combination with a PD-1 inhibitor and induces integrated innate and adaptive antitumorigenic immune responses. Tumor growth inhibition following subcutaneous implantation of **(A)** MC38 cells, **(B, C)** E.G7-OVA cells, or **(D–F)** MB49 cells. **(A)** hVISTA-KI mice were dosed with 10 mg/kg of KVA12.2a (WT, mIgG2a) or isotype control (3x/week) and/or 5 mg/kg of anti-mPD-1 (2x/week). Data are shown as means ± SEM (n=8). **(B, D)** hVISTA-KI mice were dosed with 30 mg/kg of KVA12.2a, KVA12.2a-LALA, or isotype control (2x/week). Data are shown as means ± SEM (n=10). **(C)** hVISTA-KI mice were dosed with 20 mg/kg of KVA12.1 (WT, hIgG1), KVA12123 (YTE, hIgG1), KVA12.4 (WT, hIgG4), or isotype controls (2x/week). Data are shown as means ± SEM (n=8). **(E)** hVISTA-KI mice were dosed with 30 mg/kg of KVA12.1 (WT, hIgG1), KVA12.4 (WT, hIgG4), or isotype controls (2x/week). Data are shown as means ± SEM (n=8). **(F)** hVISTA-KI mice were dosed with 20 mg/kg of KVA12123 (YTE, hIgG1), 5mg/kg of anti-mPD-1, or isotype control alone or in combination (2x/week). Data are shown as means ± SEM (n=8). **(G)** Percentages of tumor-infiltrating cells were analyzed using hVISTA-KI mice treated with 20 mg/kg KVA12123 or hIgG1. Immune flow analysis of extracted MB49 tumors on Day 12 (24 hours after the 3^rd^ dose) is shown: CD4+ T cells (CD45+, CD3+, CD4+), CD4+ effector memory T cells (CD45+, CD3+, CD4+, CD44+, CD62L-), CD8+ T cells (CD45+, CD3+, CD8+), CD8+ effector memory T cells (CD45+, CD3+, CD8+, CD44+, CD62L-), NK cells (CD45+, CD3-, NK1.1+), M1-like macrophages (CD45+, CD11b+, F4/80+, Ly6G-, Ly6C low, MHCII+), granulocytic gMDSCs (CD45+, CD11b+, Ly6G+, Ly6C low, F4/80-, MHCII−), and classical dendritic cells (cDC: CD45+, CD11C+, MHCII+). Data are shown as means ± SD (n=8). P-value was obtained by unpaired t-test. *P < 0.05, **P < 0.01, ***P < 0.001.

Next, we examined the antitumor activity of our lead mAb (KVA12) formatted on a mouse IgG2a backbone, KVA12.2a, in the hVISTA-KI C57Bl/6 mice implanted with MC38 cells. KVA12.2a was used to mimic the effector function of human IgG1 in mice. KVA12.2a (10 mg/kg, twice weekly) showed significant TGI in the MC38 tumor model as a single agent compared to isotype control with 42% TGI. When KVA12.2a was administered in combination with an anti-mPD-1 (5 mg/kg, twice weekly), efficacy increased to 70% TGI, while the anti-mPD-1 alone had a 32% TGI, indicating synergy between the two mechanisms (**Figure 6A**).

To evaluate the role played by a functional Fc in the mechanism of action of KVA12.2a *in vivo*, we generated a double LALA mutation (L234A, L235A), which strongly reduced the antibody’s effector function. KVA12.2a and the KVA 12.2a-LALA were evaluated in the E.G7-OVA (thymoma) and MB49 (bladder cancer) tumor models. Human VISTA-KI mice were subcutaneously implanted with either of these tumor cell types and received 30 mg/kg KVA12.2a or KVA12.2a-LALA twice a week for three weeks (**Figures 6B, D**). Both antibodies in the E.G7-OVA model considered a hot tumor, showed similar tumor growth inhibition with 75% and 68% TGI for KVA12.2a and KVA12.2a-LALA, respectively. In the MB49 tumor model, considered a cold tumor, KVA12.2a demonstrated significant tumor growth inhibition as a single agent compared to isotype control with 66% TGI, while KVA12.2a-LALA mutation had minimal TGI. These results confirm previous work indicating that an anti-VISTA antibody with an effector Fc function is needed for strong anti-tumor efficacy in cold solid tumors like MB49 or MC38 (33). The Fc effector function is less crucial in an immunoreactive hematological tumor like E.G7-OVA. Next, the fully human antibodies KVA12.1 (WT, hIgG1 WT), KVA12123 (YTE, hIgG1), and KVA12.4 (WT, hIgG4) were evaluated in the E.G7-OVA tumor model (**Figure 6C**). Human VISTA-KI mice received 20 mg/kg of KVA12.1, KVA12123, KVA12.4 or isotype controls (hIgG1 or hIgG4) twice a week for three weeks. The results showed that KVA12.1 and KVA12123, both formatted on an hIgG1 backbone, exhibit strong single-agent activity compared to isotype control with 48% TGI, while KVA12.4 (WT, hIgG4) demonstrated almost no TGI. Similar results were obtained using the MB49 tumor model (**Figure 6E**). We also tested KVA12.1 (WT, hIgG1) at 3, 10, or 30 mg/kg in the MB49 model and showed a dose-response compared to isotype control with 5%, 24%, and 55% TGI respectively in VISTA-KI mice (**Figure S8**). Based on these results and the results obtained with wild type or LALA mutation, we selected our lead clinical candidate KVA12 formatted on an IgG1 backbone and inserted a YTE triple mutation to extend the half-life of the antibody in humans and potentially reducing its immuno-reactivity while preserving the necessary Fc effector function.

We then selected a suboptimal dose of KVA12123 (20 mg/kg) to test its efficacy as a single agent and in combination with an anti-mPD-1 in MB49 (**Figure 6F**). Consistent with what was previously observed in the MC38 tumor model using KVA12.2a, our clinical candidate KVA12123 alone induced 45% TGI and, in combination with anti-mPD-1, demonstrated a further increased inhibition with 89% TGI. In this combination group, 4 out of 8 mice were complete regressors. To analyze the immune response that was taking place in the tumor microenvironment during treatment with KVA12123, we collected MB49 tumors to analyze tumor associated myeloid and lymphoid cells (**Figure 6G**). We observed a significant increase in the frequency of M1-like macrophages (CD45+, CD11b+, F4/80+, Ly6G-, Ly6C low, MHCII+) and classical DCs (CD45+, CD11C+, MHCII+) and a decrease in the frequency of granulocytic gMDSCs (CD45+, CD11b+, Ly6G+, Ly6C low, F4/80-, MHCII−) in the TME of mice treated with KVA12123 compared to the isotype control. We also observed a statistically significant increase of CD4+ T cells infiltrating the tumor with an effector memory phenotype (CD44+, CD62L-) consistent with the induction of a strong and long-lasting antitumor response. NK cells were also increased in MB49 tumors after treatment with KVA12123. These results suggest that VISTA blocking with KVA12123 mAb leads to a decrease in immunosuppressive cells with a reduction of gMDSCs and an enrichment of pro-inflammatory M1-like macrophages. KVA12123 also contributes to tumor antigen cross-presentation with an increase of in-migrating cDC associated with the recruitment of inflammatory effector NK cells and T cells in the TME that contribute to an effective anti-tumor response. KVA12123 treatment induces a clear shift from an immunosuppressive to a proinflammatory TME.

### 3.11 KVA12123 is well tolerated in preclinical toxicology studies

KVA12123 mAb was evaluated using male and female cynomolgus monkeys in GLP-compliant toxicology studies. Each cohort received an IV bolus injection of KVA12123 every seven days at 10 mg/kg, 30 mg/kg, or 100 mg/kg, followed by a 4-week recovery period. KVA12123 was well tolerated. All animals survived until the scheduled day of the necropsy (Day 29). There were no KVA12123-related clinical or injection site observations, or effects on body weight, qualitative food consumption, body temperature, visual respiration rate, hematology, coagulation, clinical chemistry, urinalysis parameters, absolute counts, or relative percentages of monocytes, NK cells, B lymphocytes, T lymphocyte subsets, IL-1ra, IL-1β, IL-2, IL-4, IL-5, IL-6, IL-8, IL-10, IL-12/23(p40), IL-13, IL-17A, MIP1β, IFNγ, TNFα, granulocyte colony stimulating factor (G-CSF), organ weight, or macroscopic or microscopic findings. No cytokine secretion associated with cytokine release syndrome (CRS) was observed (**Figure S9**). KVA12123-related dose-independent increases in plasma IL-1ra, MCP-1, and CXCL-10 concentrations were observed in most animals at ≥ 10 mg/kg on Day 1 (6 or 24 hrs postdose). Increases were observed of similar or greater magnitude in plasma IL-1ra and MCP-1 and of lesser magnitude CXCL-10 on Day 22 (6 or 24 hrs postdose). The increases peaked in IL-1ra and MCP-1 on Day 22 (6 hrs postdose) and in CXCL-10 on Day 1 (6 hrs postdose) and trended towards baseline by Day 1 (24 hrs postdose) and Day 22 (24 hrs postdose). All IL-1ra, MCP-1, and CXCL-10 concentrations either returned to predose values or were within the range of control animals on Day 50. Anti-drug antibodies were present in 100%, 80%, and 40% of animals after administering KVA12123 at 10, 30, and 100 mg/kg, respectively. The highest tested dose, 100 mg/kg, was determined as the no-observed-adverse-effect level (NOAEL) for KVA12123 mAbs administered once weekly by i.v. slow bolus injection in cynomolgus monkeys.

### 3.12 KVA12123 demonstrates reduced ADCC activity with no abnormal cytokine release

**Figure 7:**
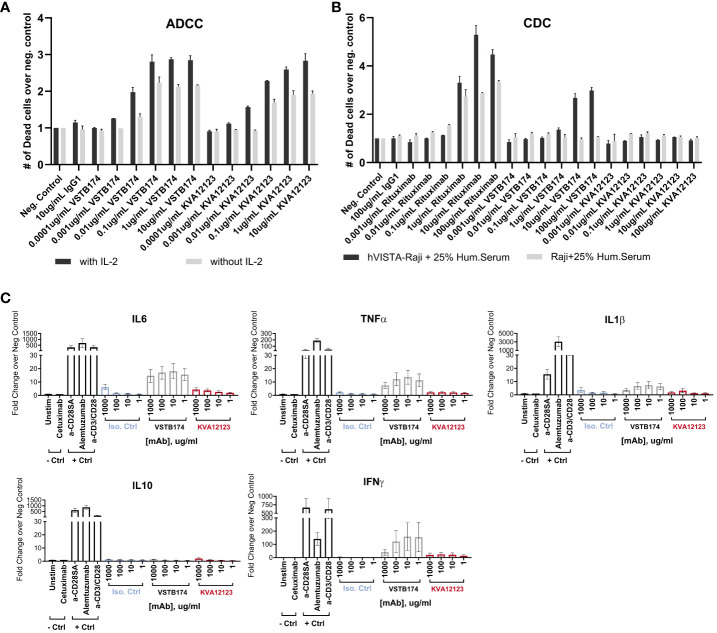
KVA12123 mAb shows reduced ADCC and no detectable CDC activity compared to VSTB174, and no evidence of CRS-associated cytokine induction. **(A)** KVA12123-mediated ADCC activity using human PBMCs and Raji cells expressing hVISTA with (dark grey) or without IL-2 (light grey) treatment. Data are shown as means ± SD (n=3) for one representative healthy donor. Six healthy donors were evaluated. **(B)** KVA12123-mediated CDC activity was measured using Raji cells with or without hVISTA expression. Data are shown as means ± SD (n=3). **(C)** Effect of KVA12123 mAb on human whole blood cytokine secretion from eleven healthy donors after 24 hours of incubation (n=2 for each donor). Data were normalized using the assay negative control. Data are shown as means ± SEM.

Since KVA12123 was engineered with an FcRn affinity-enhancing YTE mutation, we tested the effect of this mutation on ADCC and CDC. It has been reported that Rituximab containing YTE had no detectable CDC activity and a slightly reduced ADCC activity (34). Similarly, we observed that the ADCC activity of KVA12123 was reduced when compared to VSTB174 (**Figure 7A** and **Table S8**) with or without IL-2 stimulation, while CDC was ablated entirely (**Figure 7B**). We also evaluated the ability of KVA12123 to trigger non-specific cytokine release. Cytokine levels of IFNγ, IL-6, TNFα, IL-1β, IL-2, and IL-10 were measured in a human whole blood assay from eleven healthy male and female donors. Incubation of whole blood with an anti-CD28 (clone ANC28.1/5D10) super-agonist or with alemtuzumab (anti-CD52) positive controls resulted in the robust release of TNFα, IL-6, and IL-1β, cytokines associated with CRS (35, 36). While VSTB174 significantly induced IL-6 and TNFα cytokine secretion, KVA12123 did not elicit any significant cytokine release in whole blood after 24 hours of incubation over a broad range of concentrations from 1 to 1000 μg/ml. Together these results collected in human whole blood demonstrate that KVA12123 presents a low risk for immunotoxicity caused by cytokine release.

## 4 Discussion

Inhibition of specific immune checkpoint proteins of the B7/CD28 family like programmed cell death protein-1 (PD-1) and its ligand PD-L1, cytotoxic T-lymphocyte antigen-4 (CTLA-4), and more recently LAG-3 using monoclonal antibodies has revolutionized treatment for cancer patients with advanced or metastatic tumors. However, these therapies work only for a limited set of indications and, in some cases, with patients experiencing relapse after an initial response (2, 3). PD-1, CTLA-4, and LAG-3 are three important targets expressed primarily on T cells. Unfortunately, these T cells are either non-functional, fully exhausted, or even absent in cold tumors. Cold tumors generally do not respond to existing checkpoint inhibitors. Therefore, an orthogonal approach needs to be taken in patients experiencing this lack of response. Until recently, innate immune cells were almost ignored as potential targets of interest in the antitumor response, even though they mediate the first line of the immune response, providing complementary and non-overlapping functions to the adaptive response. V-domain immunoglobulin suppressor of T-cell activation (VISTA) mainly expressed on the innate immune cell populations, especially myeloid cells, controls immune homeostasis by mechanisms distinct from PD-1, CTLA-4, or LAG-3 (37). VISTA is a strong inhibitor of T cell activation and cytokine production (8). We demonstrated that VISTA blockade with the monoclonal antibody KVA12123 decreases immuno-suppression mediated by myeloid cells, activates NK cells, and promotes memory T cell infiltration in the TME. This is associated with a strong single-agent anti-tumor activity of KVA12123 in multiple tumor models, which is amplified in combination with an anti-PD-1 mAb.

KVA12123 is a fully human immunoglobulin (Ig)G1-kappa monoclonal antibody that binds specifically to the VISTA-ECD at sub-nanomolar concentrations with a fast on-rate and slow off-rate indicating that KVA12123 binds quickly and tightly to human and cynomolgus VISTA. KVA12123 does not bind to other members of the B7 family. We have shown that KVA12123 blocks VISTA interaction with its putative endogenous ligands. Initially, three ligands were described to interact with VISTA at neutral pH: VSIG-3, VSIG-8, and LRIG-1 (26–28). More recently, Syndecan-2 and Galectin-9 have also been demonstrated to interact with VISTA (38, 39). Another ligand, PSGL-1 also interacts with VISTA but only at acidic pH (18). An acidic environment is frequently observed in tumors due to the lack of oxygen (40). In addition, lactic acid, a metabolite of glycolysis, can also accumulate in the TME, leading to reduced extracellular pH (41). In these conditions, VISTA can potentially interact with specific ligands like PSGL-1. Therefore, different ligands may engage VISTA under various physiological and pathological conditions. Some groups have focused their VISTA targeting strategies by designing antibodies that bind to a histidine-rich cluster in the VISTA extracellular domain (7). These histidines are protonated at acidic pH allowing a unique interaction with PSGL-1 (18). One can hypothesize that targeting VISTA with an antibody that recognizes its target only in the acidic tumor microenvironment may improve the pharmacokinetics of the antibody by reducing target-mediated drug disposition and potentially increasing its efficacy. However, pH levels of the tumor microenvironment can vary across the same tumor from neutral to acidic depending on the level of hypoxia, resulting in low efficacy of pH-dependent anti-VISTA antibodies. Furthermore, Spitzer et al. (42) have demonstrated that effective cancer immunotherapies, while inducing immune activation in the tumor in the initial phase, only peripheral immune cells with sustained proliferation and activation are required for tumor rejection and eradication, with a key role played by a subset of CD4+ T cells. These CD4+ T cells confer protection against new tumors. This demonstrates the critical impact of a systemic immune response that drives tumor rejection. Secondary lymphoid organs are critical sites where activated dendritic cells will prime those T cells in the periphery and where immune cell interactions and activations are taking place at neutral pH (42).

We demonstrated that KVA12123 blocks the interaction of VISTA with its putative ligands and masks a major epitope involved in ligand binding at neutral and acidic pH (**Figure 3A**). We determined that KVA12123 interacts with amino acids Y37, R54, V117, and R127 of VISTA extracellular domain using the site-directed mutagenesis on surface-exposed amino acid residues. Primarily, KVA12123 interacts with an arginine at position 54 located in the VISTA C-C’ loop domain. The C-C’ loop is unique to VISTA and is not present in other members of the B7 family. It also carries key residues essential for VISTA interaction with multiple ligands such as VSIG-3 and LRIG-1 (6). The C-C’ loop is flexible and can potentially interact with distal parts of the molecule. Then, KVA12123 also binds to a region of the molecule adjacent to the histidine-rich domain, on an arginine located on a beta-sheet at position 127, preventing PSGL-1 binding to VISTA at acidic pH (**Figure S5**). Two other amino acids are crucial to stabilizing the interaction of KVA12123 with VISTA; a valine at position 117, which in the 3D structure of the molecule is close to R127, and a tyrosine at position 37, which is in the proximity of the C-C’ loop. The aromatic side chain of the arginine at position 54 can potentially interact with the side chain of the tyrosine 37 and possibly stabilize the molecule by electrostatic interaction. Therefore, the unique epitope of KVA12123 encompasses R54 and R127, the two primary amino acids, supplemented by V117 and Y37, which stabilize the interaction of the KVA12123 antibody with VISTA. The KVA12123 epitope is distinct from three other clinical stage anti-VISTA antibodies, VSTB174 (6), HMBD-002 (32), and SNS-101 (43), which either bind to the C-C’ loop of the molecule (VSTB174 and HMBD-002) or the histidine-rich domain (SNS-101). This unique epitope favors the ability of KVA12123 to block VISTA interaction with the different VISTA ligands. This epitope most likely contributes to the unique pharmacokinetic proprieties of KVA12123.

During our screening process, we observed that KVA12.1 was one of the few antibodies that exhibited an extended half-life in human VISTA-KI mice as well as in non-human primates. To further improve the pharmacokinetics of KVA12.1, we introduced the M252Y/S254T/T256E YTE mutation, that has been shown to increase FcRn binding affinity and antibody recycling (22). We showed that this mutation significantly reduced the clearance and improved the half-life of KVA12123 when compared to KVA12.1 in non-human primate PK studies (**Figure 2A**, **Tables S3** and **S4**). The YTE mutation increased the binding affinity of KVA12123 to FcRn greater than 5-fold compared to KVA12.1 WT (**Figure 2D**). It is known that increased FcRn binding reduces the impact of target-mediated metabolism, driven by the expression of VISTA on a large population of immune cells in the central compartment (e.g. monocytes, neutrophils). The YTE mutation also reduced by 4 to 7-fold the binding affinity of KVA12123 to FcγRI, FcγRIIa, and RIIIa compared to KVA12.1 WT (**Figure 2D**), and this is associated with reduced ADCC (**Figure 7A**) and an absence of CDC (**Figure 7B**). The reduced Fcγ receptor affinity and unique functional proprieties of the YTE mutation compared to the WT IgG1 mitigates the potential clinical risk from cytokine release syndrome. The release of pro-inflammatory cytokines like TNFα and IL-6, hallmarks of CRS, was observed by another group developing an anti-VISTA antibody with a wild-type IgG1 (44). This safety profile was strengthened by cynomolgus monkey studies, where KVA12123 did not induce any toxicity or cytokine release associated with CRS (**Figure S9**). An absence of IL-6 and TNFα secretion was also confirmed in whole human blood from multiple healthy donors treated *in vitro* with KVA12123 (**Figure 7C**). However, we demonstrated that even if the YTE IgG1 mutation reduced FcγR binding and ADCC, the partial Fc-effector function of KVA12123 mAb was preserved, resulting in its potent single-agent anti-tumor efficacy in cold tumor models compared to an IgG4 or an Fc-Null IgG which possess poor or no effector functions (**Figure 6**). We believe that one of the main reasons is the requirement for Fc-mediated cross-linking to NK cells, particularly through FcγRIIIa that is not abrogated by the YTE mutation. This interaction and cross-linking mediate NK cell activation and favor a potent antitumor response.

We also demonstrated that KVA12123 enhances T-cell activation and induces CD14+ monocyte activation *in vitro*, with an increase of expression of activation markers like CD80 and HLA-DR as well as an enhanced secretion of the IFN-responsive chemokine CXCL-10 (**Figure 5**). This activation of immature monocytes to myeloid cells presenting functional activation markers does not take place in the absence of NK cells, or if the antibody is engineered with an IgG4 backbone. This emphasizes the important role of NK cell-mediated cross-linking and activation through their Fc receptors in the anti-tumor response. We also showed that KVA12123 was able to reverse immunosuppression on T cells, driven by *in vitro* differentiated MDSCs. These data were further confirmed *in vivo* in hVISTA KI-mice after analyzing immune infiltrates from the MB49 tumor model after 3 doses of KVA12123. Significant increases of tumor-infiltrating CD4+ T cells with a memory phenotype as well as NK cells were observed. CD4+ T cells are necessary to maintain and sustain antitumor CD8+ CTL responses. CD4+ T cells also support and maintain pro-inflammatory cross-presenting DCs. This emphasizes the important role played by the CD4+ T cell population for an efficient and long-lasting anti-tumor immune response described previously by Spitzer et al. (42). Furthermore, after treatment with KVA12123, a switch was observed from immunosuppressive to immuno-inflammatory myeloid cells, with an enrichment of M1-like macrophages along with a reduction of granular MDSCs. This suggests that a critical remodeling is taking place in the TME, leading to inhibition of myeloid cell immunosuppression, a hallmark of cold tumors, and allowing in-migration of classical dendritic cells into the tumor to restore appropriate tumor antigen presentation to T cells.

## 5 Conclusions

We showed that KVA12123 increased immune responses by blocking signaling events mediated by VISTA and/or the cellular and molecular pathways regulated by VISTA, possibly leading to tumor growth inhibition. Moreover, tolerability and PK studies of KVA12123 performed in non-human primates have shown no KVA12123-related clinical observations or toxicities. Therefore, KVA12123 exhibits appropriate safety and PK profiles. Collectively, these data indicate that VISTA is a potent immunomodulatory protein expressed on myeloid cells in the TME and is, therefore, a relevant immunotherapy target for the treatment of cancer patients (45). Based on these observations, a phase 1/2 open-label clinical trial evaluating KVA12123 alone or in combination with pembrolizumab in patients with advanced solid tumors is currently ongoing (NCT05708950 Clinicaltrials.gov).

## Data availability statement

The original contributions presented in the study are included in the article/**Supplementary Material**. Further inquiries can be directed to the corresponding author.

## Ethics statement

Ethical approval was not required for the studies on humans in accordance with the local legislation and institutional requirements because only commercially available established cell lines were used. The animal studies were approved by the Institutional Animal Care and Use Committee (IACUC) of Charles River Laboratories and Kineta Inc. The studies were conducted in accordance with the Animal Welfare Act and the National Institute of Health guidelines. The study was conducted in accordance with the local legislation and institutional requirements.

## Author contributions

SI: Conceptualization, Formal Analysis, Funding acquisition, Methodology, Resources, Supervision, Writing – review & editing. YO: Data curation, Formal Analysis, Methodology, Supervision, Writing – original draft. KL: Data curation, Writing – review & editing. JC: Formal Analysis, Methodology, Data curation, Writing – review & editing. NE: Formal Analysis, Methodology, Data curation, Writing – review & editing. EF: Data curation, Methodology, Validation, Formal Analysis, Writing – review & editing. NK: Data curation, Methodology, Validation, Formal Analysis, Writing – review & editing. CK: Conceptualization, Data curation, Formal Analysis, Methodology, Supervision, Validation, Writing – review & editing. RL: Formal Analysis, Methodology, Validation, Writing – review & editing. DP: Conceptualization, Data curation, Formal Analysis, Methodology, Writing – review & editing. SS: Conceptualization, Data curation, Formal Analysis, Methodology, Supervision, Writing – review & editing. CT: Data curation, Formal Analysis, Methodology, Writing – review & editing. IT: Formal Analysis, Methodology, Writing – review & editing. MX: Data curation, Formal Analysis, Methodology, Writing – review & editing. TG: Conceptualization, Data curation, Formal Analysis, Funding acquisition, Methodology, Supervision, Validation, Writing – original draft.
