# Corrigendum: A highly potent anti-VISTA antibody KVA12123 - a new immune checkpoint inhibitor and a promising therapy against poorly immunogenic tumors

**DOI:** 10.3389/fimmu.2024.1365240

**Published:** 2024-01-23

**Authors:** Shawn Iadonato, Yulia Ovechkina, Kurt Lustig, Jessica Cross, Nathan Eyde, Emily Frazier, Neda Kabi, Chen Katz, Remington Lance, David Peckham, Shaarwari Sridhar, Carla Talbaux, Isabelle Tihista, Mei Xu, Thierry Guillaudeux

**Affiliations:** Kineta Inc., Seattle, WA, United States

**Keywords:** Vista, PD-1H, B7-H5, immune checkpoint inhibitor, immunotherapy, PD-1 combination therapy, poorly immunogenic tumors, tumor microenvironment immunosuppression

In the published article, there was an error. More accurate sentences and methods were provided in the Abstract and Material and Methods sections to explain the selection of our fully human monoclonal antibodies.

A correction has been made to **Abstract**, *Methods*. This sentence previously stated:

“**Methods:** Fully human monoclonal antibodies directed against VISTA were produced after immunizing humanized Trianni mice and single B cell sequencing. Anti-VISTA antibodies were evaluated for specificity, cross-reactivity, monocyte and T cell activation, Fc-effector functions, and antitumor efficacy using *in vitro* and *in vivo* models to select the KVA12123 antibody lead candidate. The pharmacokinetics and safety profiles of KVA12123 were evaluated in cynomolgus monkeys.”

The corrected sentence appears below:

“**Methods:** Fully human monoclonal antibodies directed against VISTA were produced after immunizing humanized Trianni mice and sorting and sequencing natively-linked B cell scFv repertoires. Anti-VISTA antibodies were evaluated for specificity, cross-reactivity, monocyte and T cell activation, Fc-effector functions, and antitumor efficacy using *in vitro* and *in vivo* models to select the KVA12123 antibody lead candidate. The pharmacokinetics and safety profiles of KVA12123 were evaluated in cynomolgus monkeys.”

A correction has also been made to **Materials and Methods**, **2.1 Antibody library generation**. This sentence previously stated:

**“2.1 Antibody library generation**

Fully human ScFv antibodies directed against human VISTA were generated after immunization of humanized Trianni mice and single B cell sequencing. Briefly, transgenic humanized Trianni mice were immunized with soluble human VISTA extracellular domain. B cells were isolated from spleen, lymph nodes, and bone marrow. B cells were then encapsulated into droplets with oligo-dT beads and a lysis solution to generate a DNA amplicon that encodes the scFv libraries with native pairing heavy and light Ig. The scFv libraries were then transfected into yeast cells and stained with the fluorescently labeled soluble VISTA to collect scFv with the highest fluorescent signal. Finally, deep sequencing was used to identify all clones in the pre- and post-sort populations”

The corrected sentence appears below:

**“2.1 Antibody library generation**

Fully human scFv antibodies directed against human VISTA were generated after immunization of humanized Trianni® mice and sorting natively-linked B cell scFv repertoires. Briefly, transgenic humanized Trianni mice were immunized with soluble human VISTA-His extracellular domain (R&D Systems). B cells were isolated from spleen, lymph nodes, and bone marrow. B cells were then encapsulated into droplets with oligo-dT beads and a lysis solution, followed by overlap-extension RT-PCR to generate a DNA amplicon that encodes the scFv libraries with native pairing heavy and light Ig. The scFv libraries were then transfected into yeast cells for surface display, stained with biotinylated VISTA-His protein and Streptavidin-PE conjugate (Life Tech), and scFvs binding to VISTA were sorted by FACS (BD Influx). Finally, deep sequencing (Illumina) was used to identify all clones in the pre- and post-sort populations.”

Additionally, in the published article, there was an error in the Acknowledgement statement. The correct Acknowledgement statement appears below.

**Acknowledgement Statement**

VISTA antibody library generation from Trianni mice was performed by GigaGen, Inc.; all identified variable sequences were licensed by Kineta from GigaGen. All other contributions were associated with the authors listed in this paper.

The authors apologize for these errors and state that they do not change the scientific conclusions of the article in any way. The original article has been updated.

