# Supplementary material for: A highly potent anti-VISTA antibody KVA12123 - a new immune checkpoint inhibitor and a promising therapy against poorly immunogenic tumors
Source: Front Immunol. 2023 Dec 12;14:1311658. doi: 10.3389/fimmu.2023.1311658 (PMC10751915; doi:10.3389/fimmu.2023.1311658)
Supplement: Supplementary file 1 [file DataSheet_1.pdf]

Supplemental Figure S1

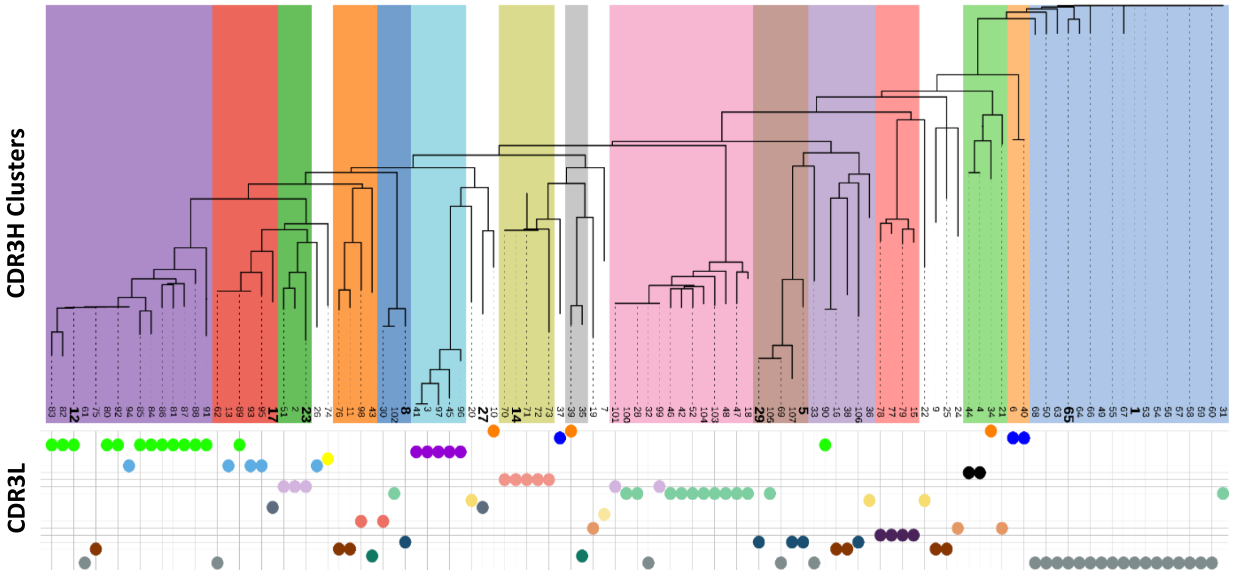

Supplemental Figure S1. 107 fully human scFv clones directed against human VISTA show high levels of sequence diversity in both heavy and light chains. Sequence analysis was performed using Clustal Omega (14) to cluster scFv sequences into clades based on similarity in the heavy and light chain CDR3 regions followed by a dendrogram representation regrouping sequences by proximal homology clades.

Supplemental Figure S2

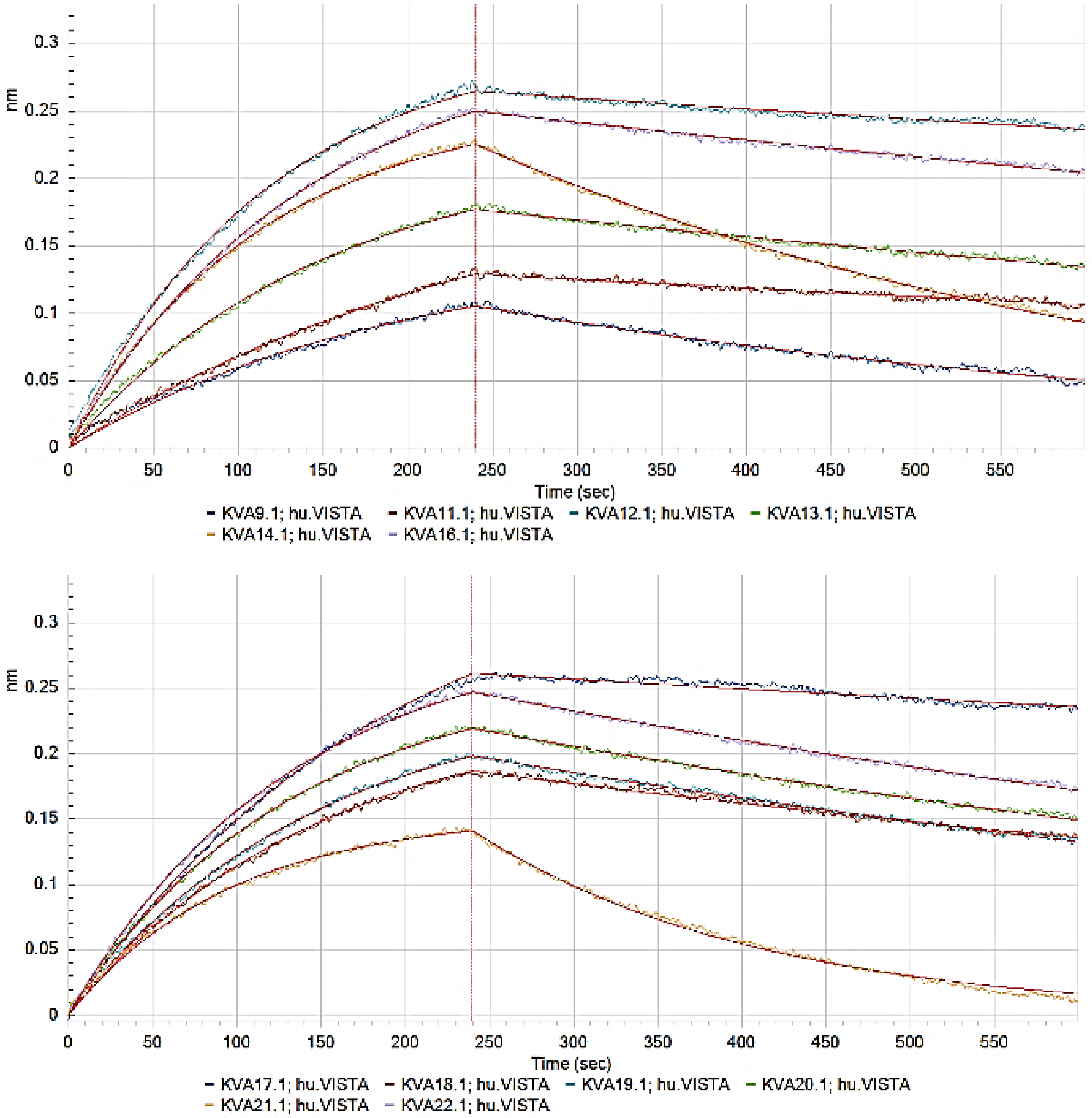

Supplemental Figure S2. Evaluation of KVA mAb binding to human VISTA by bio-layer interferometry (BLI). Representative binding sensorgrams for KVA mAb-antigen interactions were generated using Octet (ForteBio, Sartorius AG). Monomeric hVISTA-ECD was incubated with a low-density anti-hFc-captured KVA mAb biosensor for a 240-second association period and transferred to PBS for a 360-second dissociation period. A 1:1 global curve fitting analysis was performed to determine equilibrium (KD), association (ka), and dissociation (kdis) rate constants.

Supplemental Figure S3

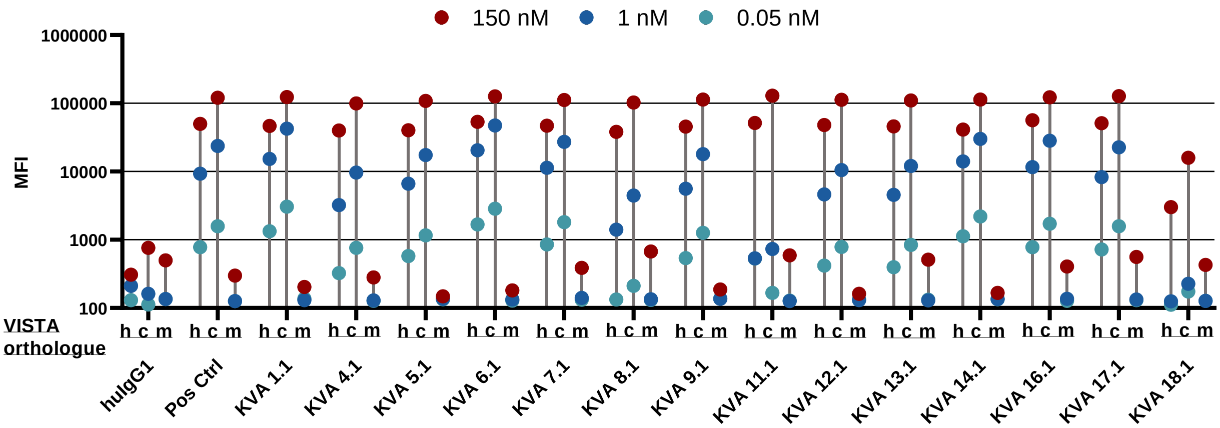

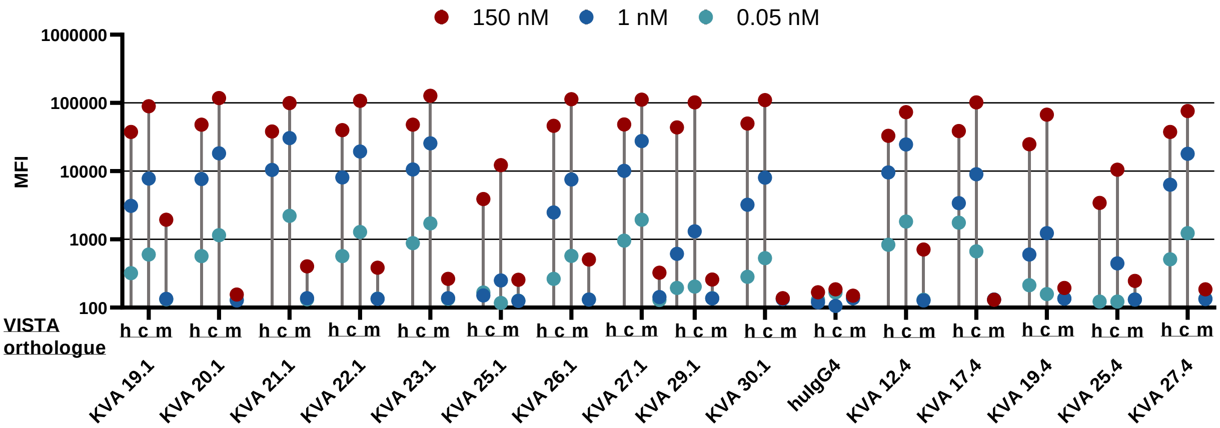

Supplemental Figure S3. KVA antibodies show potent binding to human and cynomolgus monkey VISTA but not to mouse VISTA. Human (h), cynomolgus monkey (c), or mouse (m) VISTA-expressing CHO-K1 cells were incubated with KVA mAbs at 150 nM, 1 nM and 0.05 nM concentrations. Mean fluorescence intensity levels (MFI) were determined on viable cells using flow cytometry (Thermo Fisher Attune NxT). KVA mAbs on a human IgG1 or IgG4 backbone are indicated by .1 or .4, respectively.

Supplemental Figure S4

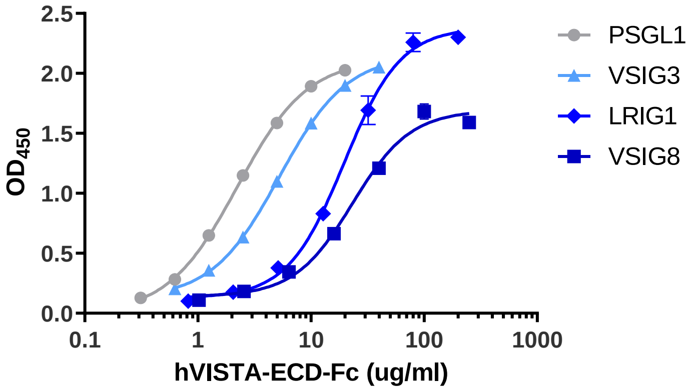

	PSGL1-VISTA	VSIG3-VISTA	VSIG8-VISTA	LRIG1-VISTA
EC50 (ug/mL)	2	5	21	19

Supplemental Figure S4. VISTA strongly interacts with VSIG3, VSIG8, or LRIG1 at pH 7.4 and with PSGL1 at pH 6.0**.** ELISA binding measurements of human VISTA interactions with its human binding partners VSIG3, VSIG8, LRIG1, and PSGL1. Data are shown as means ± SD (n=2).

Supplemental Figure S5

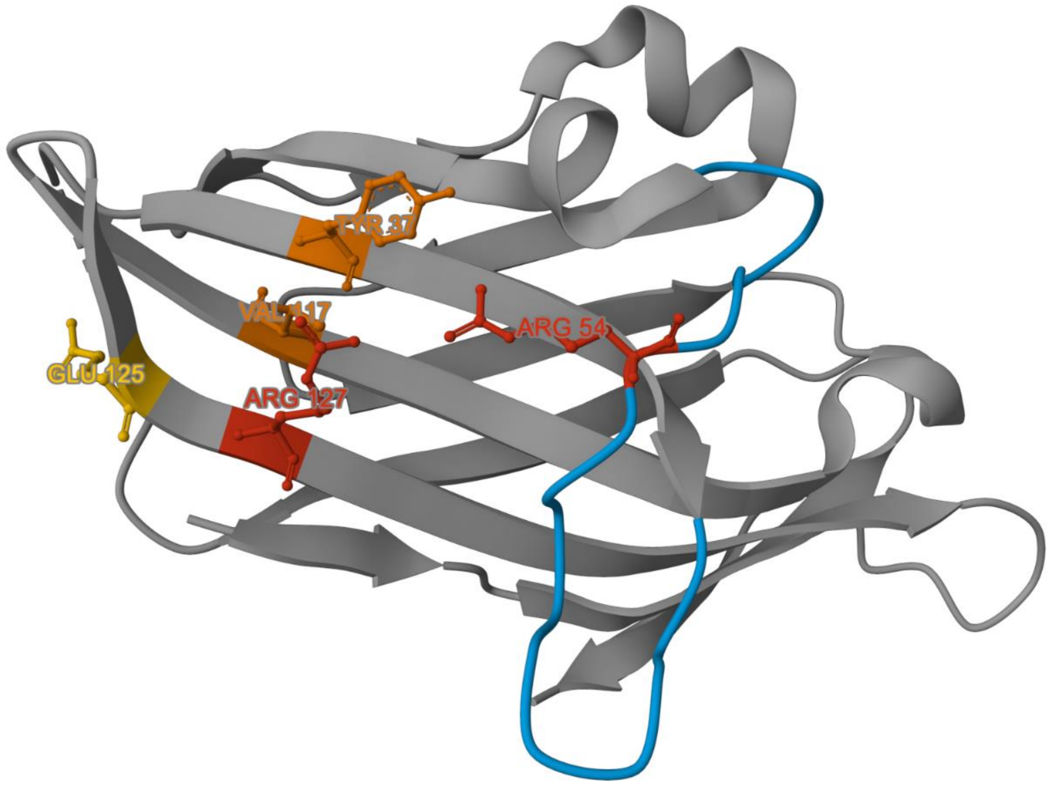

Supplemental Figure S5. VISTA-ECD crystal structure shows the predicted epitope for KVA12123 mAbs: R54 and R127 residues (red), Y37 and V117 residues (orange), and E125 residue (yellow). The VISTA C-C' loop is in blue. The image was generated using PDB ID 6OIL by the RCSB PDB (RCSB.org) (Mehta N, Maddineni S, Mathews II, Andres Parra Sperberg R, Huang PS, Cochran JR. Structure and Functional Binding Epitope of V-domain Ig Suppressor of T Cell Activation. Cell Rep. 2019 Sep;28(10):2509-2516.e5).

Supplemental Figure S6

A

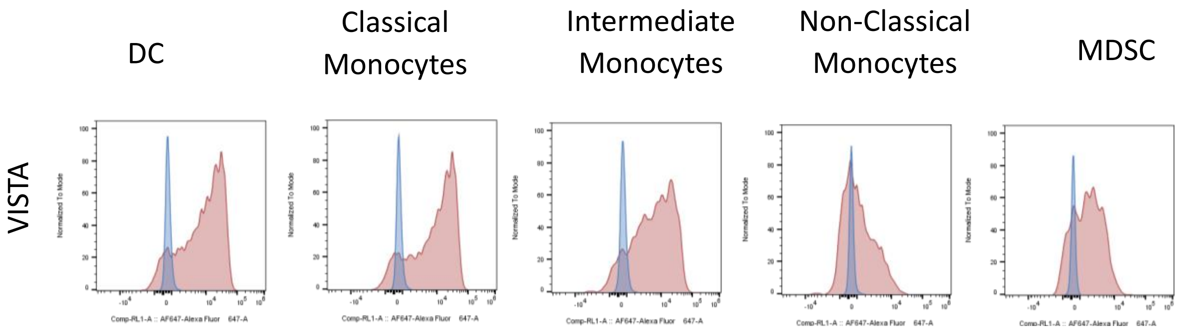

B

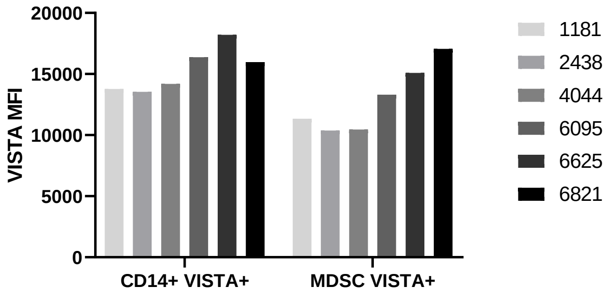

Supplemental Figure S6. Analysis of VISTA expression on CD14+ and MDSC cells. **(A)** K2EDTA-treated whole blood of a healthy human donor (Bloodworks Northwest) was stained with an antibody cocktail for myeloid cell populations (Biolegend) and labeled with anti-hVISTA KVA antibodies. Mean fluorescence intensity levels (MFI) were determined on viable cells using flow cytometry (Thermo Fisher Attune NxT). VISTA (red) and isotype control (blue) histogram overlays are shown. (**B**) Human PBMCs obtained from six healthy donors (Bloodworks Northwest) were stained with a myeloid panel (Biolegend) and analyzed by flow cytometry for VISTA expression on CD14+ monocytes and MDSCs. The six healthy donor identification numbers are 1181, 2438, 4044, 6095, 6625, and 6821.

Supplemental Figure S7

A

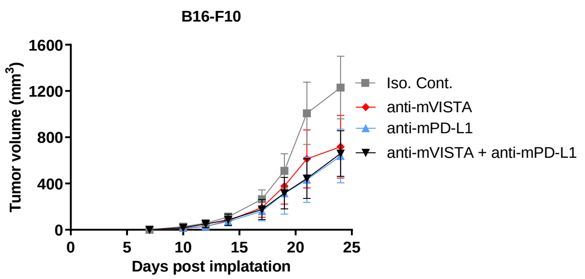

B

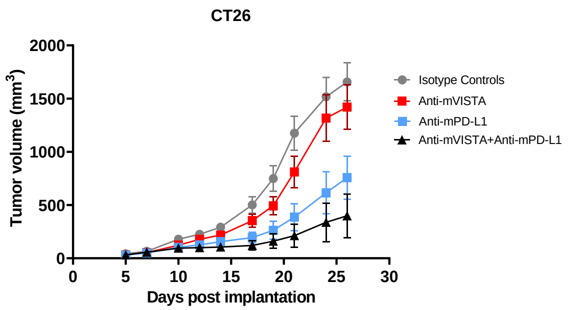

C

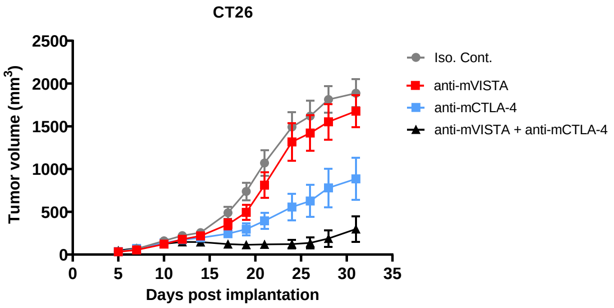

Supplemental Figure S7. The anti-mouse VISTA antibody (13F3) induces tumor growth inhibition alone or with other checkpoint inhibitors. Tumor growth inhibition following subcutaneous implantation of (A) B16-F10 cells using C57Bl/6 mice or (B, C) CT26 cells using BALB/cJ mice. (A, B) Mice were dosed with 15 mg/kg of anti-mVISTA 13F3 or isotype control and/or 10 mg/kg of anti-mPD-L1 (3x/week). (C) Mice were dosed with 15 mg/kg of anti-mVISTA 13F3 or isotype control and/or 10 mg/kg of anti-mCTLA-4 (3x/week). Data are shown as means ± SEM (n=12).

Supplemental Figure S8

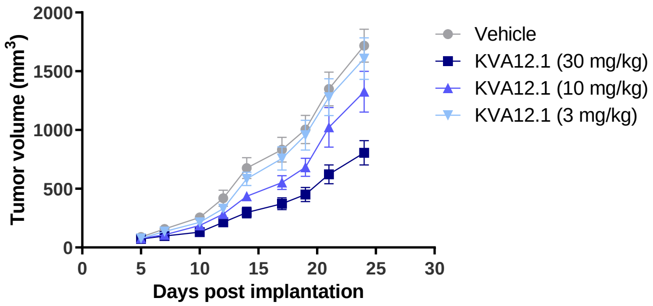

Supplemental Figure S8. KVA12.1 mAbs demonstrate the dose-dependent tumor growth inhibition in hVISTA-KI mouse transplanted with MB49 cancer cells. Animals received 3 mg/kg, 10 mg/kg, or 30 mg/kg i.p. injections of KVA12.12x/week for three weeks. Data are shown as means ± SEM (n=12).

Supplemental Figure S9

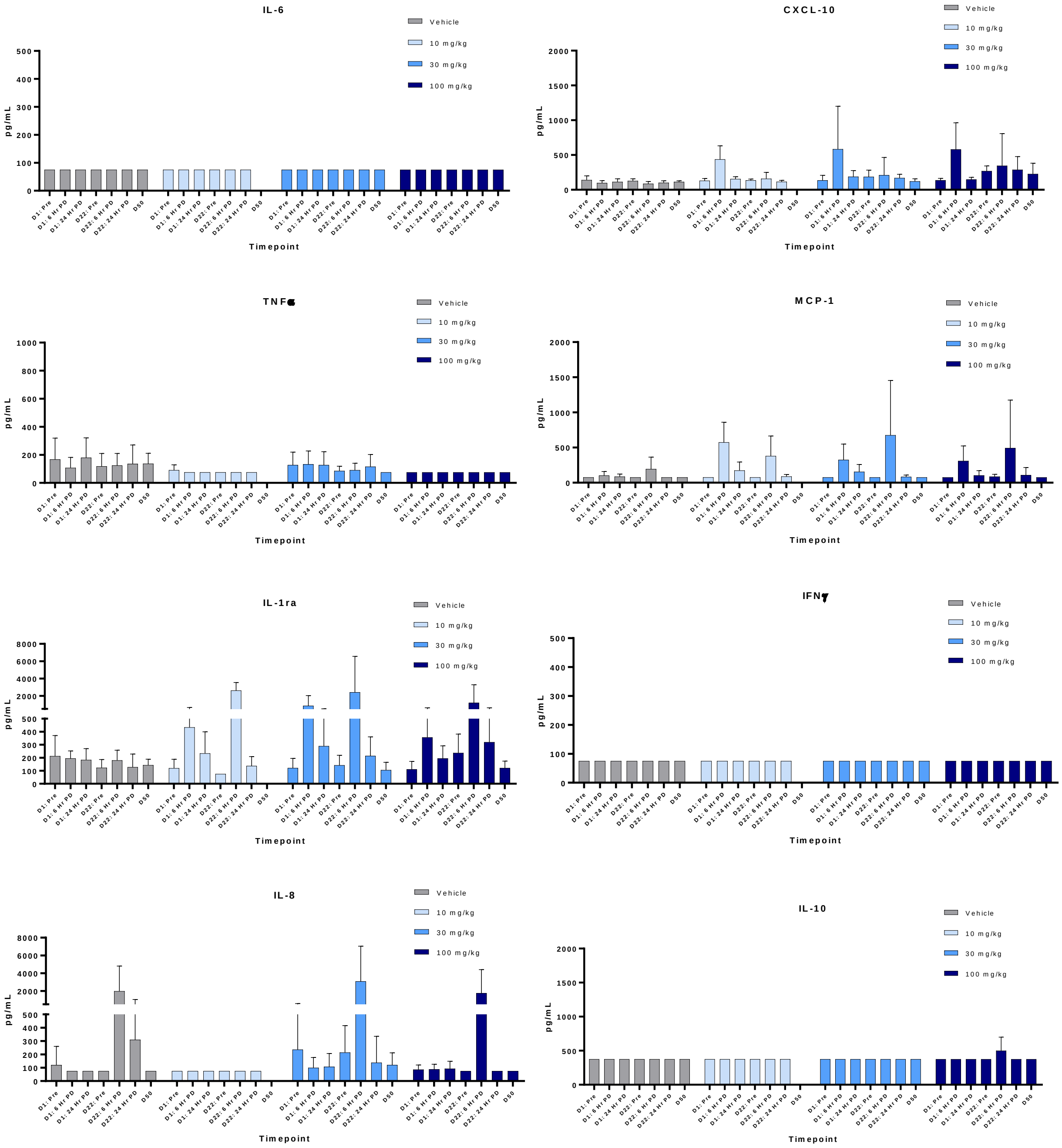

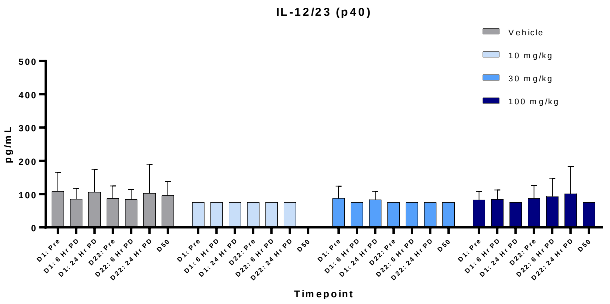
IL-12/23 (p40)
Vehicle
10 mg/kg
30 mg/kg
100 mg/kg
pg/mL
D1: Pre
D1: 6 Hr PD
D1: 24 Hr PD
D22: Pre
D22: 6 Hr PD
D22: 24 Hr PD
D50
Timepoint

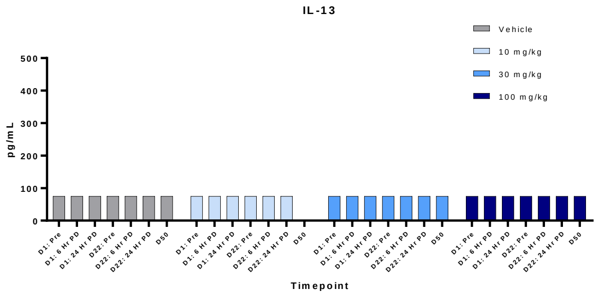
IL-13
Vehicle
10 mg/kg
30 mg/kg
100 mg/kg
pg/mL
D1: Pre
D1: 6 Hr PD
D1: 24 Hr PD
D22: Pre
D22: 6 Hr PD
D22: 24 Hr PD
D50
Timepoint

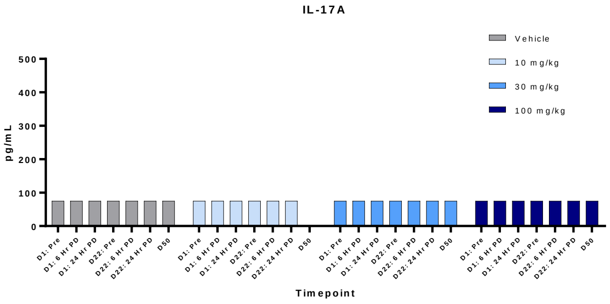
IL-17A
Vehicle
10 mg/kg
30 mg/kg
100 mg/kg
pg/mL
D1: Pre
D1: 6 Hr PD
D1: 24 Hr PD
D22: Pre
D22: 6 Hr PD
D22: 24 Hr PD
D50
Timepoint

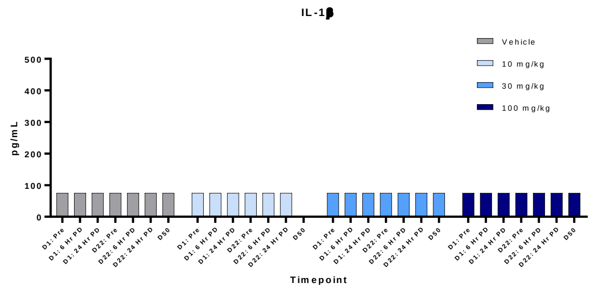
IL-1β
Vehicle
10 mg/kg
30 mg/kg
100 mg/kg
pg/mL
D1: Pre
D1: 6 Hr PD
D1: 24 Hr PD
D22: Pre
D22: 6 Hr PD
D22: 24 Hr PD
D50
Timepoint

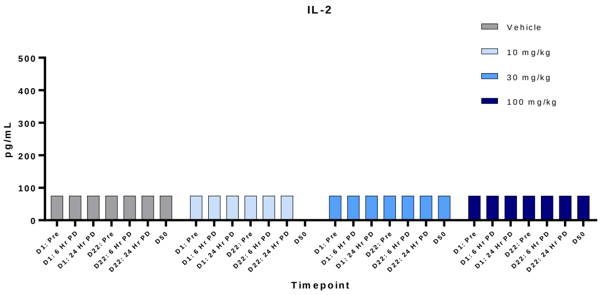
IL-2
Vehicle
10 mg/kg
30 mg/kg
100 mg/kg
pg/mL
D1: Pre
D1: 6 Hr PD
D1: 24 Hr PD
D22: Pre
D22: 6 Hr PD
D22: 24 Hr PD
D50
Timepoint

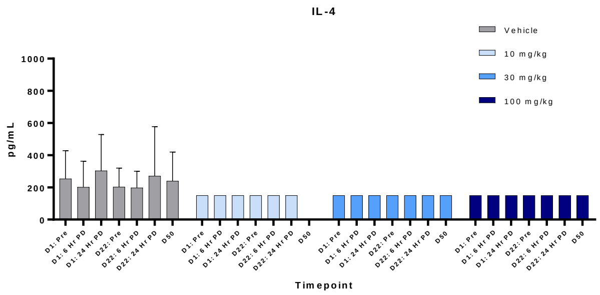
IL-4
Vehicle
10 mg/kg
30 mg/kg
100 mg/kg
pg/mL
D1: Pre
D1: 6 Hr PD
D1: 24 Hr PD
D22: Pre
D22: 6 Hr PD
D22: 24 Hr PD
D50
Timepoint

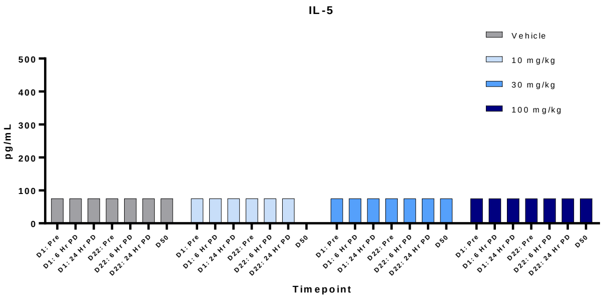
IL-5
Vehicle
10 mg/kg
30 mg/kg
100 mg/kg
pg/mL
D1: Pre
D1: 6 Hr PD
D1: 24 Hr PD
D22: Pre
D22: 6 Hr PD
D22: 24 Hr PD
D50
Timepoint

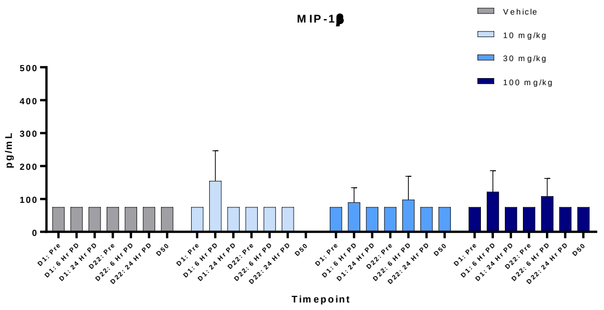
MIP-1β
Vehicle
10 mg/kg
30 mg/kg
100 mg/kg
pg/mL
D1: Pre
D1: 6 Hr PD
D1: 24 Hr PD
D22: Pre
D22: 6 Hr PD
D22: 24 Hr PD
D50
Timepoint

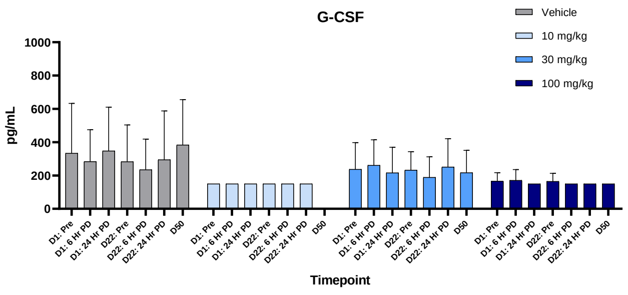
G-CSF
Vehicle
10 mg/kg
30 mg/kg
100 mg/kg
pg/mL
D1: Pre
D1: 6 Hr PD
D1: 24 Hr PD
D22: Pre
D22: 6 Hr PD
D22: 24 Hr PD
D50
Timepoint

Supplemental Figure S9. Cytokine release analysis in cynomolgus monkey preclinical toxicology study. Cytokine secretion was analyzed in serum collected from 36 cynomolgus monkeys (18 males and 18 females) dosed with KVA12123 or the vehicle control at 0, 10, 30, and 100 mg/kg via intravenous (i.v.) injection once weekly for 4 weeks. Blood was collected on Days 1 and 22 predose (Pre) or 6 and 24 hours (hr) postdose (PD) and on Day 50 (except for 10 mg/kg group). Simultaneous determination of multiple cytokine concentrations was carried out using the Luminex kits according to the manufacturer's instructions.

Table S1. ELISA screening to evaluate KVA mAbs binding to human (h), cynomolgus monkey (cyno), or mouse (m) VISTA. EC50s were calculated by using a four-parameter nonlinear regression fitting. ND stands for not determined.

Antibody	hVISTA (OD)	cynoVISTA (OD)	mVISTA (OD)	hVISTA EC_{50} (ug/mL)	hVISTA EC_{50} (nM)
KVA 1.1	2.168	0.086	0.006	0.478	3.19
KVA 4.1	2.206	0.254	0.013	0.644	4.30
KVA 5.1	2.313	1.259	0.033	0.232	1.55
KVA 6.1	1.940	0.116	0.011	0.128	0.85
KVA 7.1	2.230	0.451	0.006	0.051	0.34
KVA 8.1	1.665	1.722	0.064	0.677	4.52
KVA 9.1	1.944	0.083	0.004	1.144	7.63
KVA 11.1	2.311	0.731	0.046	0.837	5.58
KVA 12.1	2.245	0.951	0.016	0.130	0.87
KVA 13.1	1.154	0.033	0.008	1.382	9.22
KVA 14.1	1.608	0.062	0.005	1.137	7.58
KVA 16.1	2.226	0.552	0.019	0.180	1.20
KVA 17.1	2.042	0.074	0.007	0.621	4.14
KVA 18.1	2.198	1.803	0.022	ND	ND
KVA 19.1	1.667	1.511	0.149	1.687	11.25
KVA 20.1	1.961	0.064	0.005	0.488	3.25
KVA 21.1	2.119	0.091	0.028	0.788	5.26
KVA 22.1	2.229	0.145	0.017	0.206	1.37
KVA 23.1	2.247	0.171	0.013	0.397	2.65
KVA 25.1	1.383	1.116	1.254	0.240	1.60
KVA 26.1	2.251	0.299	0.011	0.870	5.80
KVA 27.1	2.381	0.197	0.009	0.306	2.04
KVA 29.1	2.290	0.686	0.036	0.739	4.93
KVA 30.1	2.126	0.102	0.022	0.901	6.01
KVA 33.1	0.008	0.005	0.006	<0.003	<0.02
KVA 34.1	0.029	0.030	0.030	<0.003	<0.02
KVA 35.1	0.028	0.032	0.026	<0.003	<0.02
KVA 36.1	0.049	0.083	0.084	<0.003	<0.02
KVA 37.1	0.032	0.051	0.045	<0.003	<0.02
KVA 38.1	0.997	0.080	0.022	2.805	18.71

KVA 39.1	0.037	0.034	0.031	<0.003	<0.02
KVA 43.1	0.011	0.018	0.018	<0.003	<0.02
KVA 48.1	0.215	0.228	0.196	ND	ND
KVA 63.1	1.736	0.050	0.014	0.150	1.00
KVA 65.1	2.199	0.092	0.003	0.074	0.49
KVA 66.1	0.070	0.048	0.031	<0.003	<0.02
KVA 74.1	1.211	0.064	0.016	0.452	3.02
KVA 77.1	1.298	1.182	0.051	ND	ND
KVA 81.1	2.169	0.406	0.039	0.314	2.09
KVA 82.1	0.092	0.096	0.071	<0.003	<0.02
KVA 91.1	2.332	0.913	0.089	0.073	0.49
KVA 95.1	0.995	0.837	0.210	ND	ND
KVA 96.1	2.402	0.983	0.049	0.030	0.20
KVA 98.1	0.222	0.207	0.051	0.085	0.56
KVA 102.1	0.130	0.194	0.097	ND	ND
KVA 106.1	2.168	0.086	0.006	ND	ND

Table S2. Evaluation of KVA mAbs binding to human (h) or cynomolgus monkey (cyno) VISTA-ECD by bio-layer interferometry (BLI). Monomeric VISTA-ECD was incubated with a low-density anti-hFc-captured KVA mAb biosensor for a 240-second association period and transferred to PBS for a 360-second dissociation period. A 1:1 global curve fitting analysis was performed to determine equilibrium (KD), association (ka), and dissociation (kdis) rate constants. BLQ stands for below the limit of quantification. ND stands for not determined.

Antibody	hVISTA Response	hVISTA KD (M)	hVISTA Kon (1/Ms)	hVISTA Kdis (1/s)	cynoVISTA Response	cynoVISTA KD (M)	cynoVISTA Kon (1/Ms)	cynoVISTA Kdis (1/s)
KVA1.1	0.2985	2.86E-09	1.34E+05	3.82E-04	0.2114	1.60E-09	2.12E+05	3.38E-04
KVA4.1	0.097	2.29E-08	4.69E+04	1.08E-03	ND	ND	ND	ND
KVA5.1	0.2414	2.44E-09	9.57E+04	2.33E-04	0.1995	1.47E-09	1.41E+05	2.07E-04
KVA6.1	0.2933	8.25E-09	1.77E+05	1.46E-03	ND	ND	ND	ND
KVA7.1	0.284	1.18E-08	1.56E+05	1.85E-03	0.2054	9.36E-09	2.48E+05	2.32E-03
KVA8.1	0.0764	8.75E-08	4.34E+04	3.80E-03	ND	ND	ND	ND
KVA9.1	0.106	3.31E-08	6.21E+04	2.05E-03	ND	ND	ND	ND
KVA11.1	0.1296	8.26E-09	6.50E+04	5.37E-04	0.1018	4.46E-08	2.30E+04	1.02E-03
KVA12.1	0.2681	1.88E-09	1.68E+05	3.15E-04	0.2223	9.36E-10	1.84E+05	1.72E-04
KVA13.1	0.1762	6.47E-09	1.18E+05	7.64E-04	0.157	1.32E-08	8.10E+04	1.07E-03
KVA14.1	0.2253	1.90E-08	1.29E+05	2.45E-03	ND	ND	ND	ND
KVA16.1	0.2494	4.20E-09	1.34E+05	5.60E-04	0.2263	1.84E-09	1.72E+05	3.17E-04
KVA17.1	0.2527	2.83E-09	1.01E+05	2.87E-04	0.2082	2.48E-09	1.57E+05	3.88E-04
KVA18.1	0.1835	7.57E-09	1.17E+05	8.88E-04	ND	ND	ND	ND
KVA19.1	0.197	9.66E-09	1.16E+05	1.12E-03	0.129	2.74E-08	8.14E+04	2.23E-03
KVA20.1	0.2189	8.24E-09	1.30E+05	1.07E-03	ND	ND	ND	ND
KVA21.1	0.1417	6.57E-08	9.05E+04	5.94E-03	ND	ND	ND	ND
KVA22.1	0.2464	7.70E-09	1.31E+05	1.01E-03	0.2421	9.42E-09	1.41E+05	1.33E-03
KVA23.1	0.2755	1.46E-09	1.45E+05	2.11E-04	0.2589	1.13E-09	1.84E+05	2.08E-04
KVA25.1	0.0146	4.65E-06	1.62E+03	7.54E-03	ND	ND	ND	ND
KVA26.1	0.1341	1.63E-08	7.65E+04	1.24E-03	ND	ND	ND	ND
KVA27.1	0.2918	1.29E-09	1.70E+05	2.20E-04	0.3136	2.60E-09	1.55E+05	4.02E-04
KVA29.1	0.1569	5.16E-09	9.65E+04	4.98E-04	0.1587	8.75E-09	8.10E+04	7.09E-04
KVA30.1	0.079	7.62E-08	1.84E+05	1.40E-02	ND	ND	ND	ND
KVA 63.1	0.2624	4.20E-09	1.83E+05	7.67E-04	0.1484	1.93E-08	7.69E+04	1.48E-03
KVA 65.1	0.3343	2.13E-09	2.29E+05	4.86E-04	0.2315	6.79E-09	1.45E+05	9.81E-04
KVA 74.1	ND	ND	ND	ND	0.0938	1.29E-07	1.21E+04	1.55E-03

KVA 77.1	ND	ND	ND	ND	-0.0434	BLQ	BLQ	BLQ
KVA 91.1	ND	ND	ND	ND	0.1003	2.24E-08	2.68E+04	6.00E-04
KVA 96.1	ND	ND	ND	ND	0.0429	8.09E-06	2.41E+03	1.95E-02
KVA 98.1	ND	ND	ND	ND	0.0181	5.91E-06	6.10E+02	3.60E-03

Table S3. Pharmacokinetic parameters of KVA mAbs in plasma of hVISTA-KI mice following a single i.p. infusion. Plasma was sampled at 2, 4, 8, 12, 24, 48, and 72-hour time points. PK parameters were calculated using non-compartmental analysis after extravascular input using PKSolver 2.0 software (Zhang Y, Huo M, Zhou J, Xie S. PKSolver: An add-in program for pharmacokinetic and pharmacodynamic data analysis in Microsoft Excel, Computer Methods and Programs in Biomedicine (2010), 99:3, 306-314).

Parameter		Unit	KVA 14.1 10 mg/kg	KVA 27.1 10 mg/kg	KVA 23.1 10 mg/kg	KVA 17.1 10 mg/kg	KVA 1.1 10 mg/kg	KVA 5.1 10 mg/kg	VSTB 174 10 mg/kg	KVA 12.2a 10 mg/kg	KVA 12.1 10 mg/kg	KVA 12.1 30 mg/kg	KVA 12.1 100 mg/kg
Peak plasma concentration	C_{max}	ug/ml	91	46	107	85	77	98	92	116	115	645	1514
Last plasma concentration	C_{last}	ug/ml	0.01	1	26	0.02	3	58	0.003	0.03	24	6	156
Time of peak plasma concentration	T_{max}	hr	2	4	2	4	2	2	4	8	4	4	4
Time of last plasma concentration	T_{last}	hr	8	12	12	12	12	8	24	48	24	72	72
Elimination half-life	$T_{1/2}$	hr	0.4	1.6	3.9	0.7	2.0	8.8	1.3	3.4	9.4	9.6	32.9
Area under the curve	AUC_{0-t}	ug/ml*hr	181	249	822	440	361	495	1046	1831	1457	11008	37414

Table S4. Pharmacokinetic parameters for KVA12.1 and KVA12123 in serum of female cynomolgus monkeys following a single i.v. infusion. Serum was sampled pre-dose and at 0.083, 1, 6, 12, 24, 72, 96, 144, 168, 216, 264, 336, and 672-hour time points post-dose. Pharmacokinetic parameters were calculated using Phoenix WinNonlin v8.3 (Certara, Princeton, NJ) with non-compartmental analysis using an i.v. bolus model.

Parameter		Unit	KVA12.1 30mg/kg	KVA12123 30mg/kg	KVA12.1 100mg/kg	KVA12123 100mg/kg
Peak serum concentration	C_{max}	ug/ml	1010	1029	3110	3230
Last serum concentration	C_{last}	ug/ml	0.2	0.1	13	0.2
Time of peak serum concentration	T_{max}	hr	0.083	0.083	0.083	0.083
Time of last serum concentration	T_{last}	hr	168	264	336	672
Elimination half-life	Initial $T_{1/2}$	hr	29.9	47.6	103	165
Elimination half-life	Beta $T_{1/2}$	hr	8.3	11.0	28.0	33.0
Area under the curve	AUC_{0-t}	ug/ml*hr	31900	42600	185000	310000

Table S5. KVA12123 (YTE) demonstrates a 9-fold higher affinity to FcRn than KVA12.1 (WT) at pH 6.0. Fitted association and dissociation curves were generated using Octet (FortéBio, Sartorius AG). A 1:1 global curve fitting analysis was performed to determine equilibrium (KD), association (ka) and dissociation (kdis) rate constants.

	FcRn KD (M) at pH 6.0	FcRn Ka (1/Ms) at pH 6.0	FcRn Kdis (1/s) at pH 6.0
**KVA12123 (YTE, IgG1)**	8.4E-08	2.3E+05	1.9E-02
**KVA12.1 (WT, IgG1)**	7.6E-07	4.7E+04	3.7E-02
**Fold Change from WT**	9	5	2

Table S6. Evaluation of VISTA-ECD mutations on KVA12.1, KVA12123, or VSTB174 antibody binding by ELISA. EC50s were calculated by using a four-parameter nonlinear regression fitting. ND stands for not determined.

hVISTA Mutations	KVA12.1 EC_{50} (ug/mL)	KVA12123 EC_{50} (ug/mL)	VSTB174 EC_{50} (ug/mL)	IgG1 EC_{50} (ug/mL)
None (WT)	0.12	0.15	0.06	>100
R54A, F62A, Q63A	0.14	0.12	>100	>100
H66A, H68A, H72A, H93A, H94A, H121A, H122A, and H123A	0.14	ND	0.08	>100
H121A, H122A, and H123A	0.16	ND	0.1	>100
H66A, H68A	0.15	ND	0.1	>100
N59A, T61A, D64A	0.47	ND	0.1	>100
H79A, D80A, L81A	0.20	ND	0.1	>100
Q83A, R84A, H85A	0.20	ND	0.1	>100
T35A, I119A, S124A	0.20	ND	>100	> 100
T35A	0.24	ND	0.1	> 100
I119A	0.2	ND	0.07	> 100
S124A	0.19	ND	0.07	> 100
T35A, I119A	0.18	ND	0.05	> 100
I119A, S124A	0.19	ND	74	> 100
T35A, S124A	0.20	ND	0.09	> 100
L115A, H129A	0.19	ND	0.1	>100
Y37A, V117A, R127A	56.0	>100	21	> 100
Y37A	0.19	ND	0.06	> 100
V117A	0.17	ND	0.09	> 100
R127A	0.10	ND	0.07	> 100
Y37A, V117A	0.19	ND	0.04	> 100
V117A, R127A	0.11	ND	0.08	> 100
Y37A, R127A	0.14	ND	0.06	> 100
E47A, V48A, Q49A	0.20	ND	0.1	> 100
S52A, E53A, R55A	0.22	ND	0.1	> 100
Q73A, N76A	0.19	ND	0.1	> 100
H32A, D33A, T35A	0.17	ND	0.1	> 100
E14A, L106A, L107A	0.19	ND	0.1	> 100
F36A	0.28	ND	0.08	> 100
K38A	0.28	ND	0.09	> 100
T39A	0.21	ND	0.11	> 100

E125A	0.14	ND	0.08	> 100
H122A	0.18	0.30	0.06	> 100
R54A	3	4	4.8	> 100
F62A	0.21	0.20	0.04	> 100
Q63A	0.18	0.20	0.06	> 100
R54A, R127A	> 100	> 100	> 100	> 100
F62A, R127A	0.11	0.10	0.05	> 100
Q63A, R127A	0.11	0.20	0.04	> 100

Table S7. Evaluation of VISTA-ECD mutations on KVA12.2a antibody binding by Bio-Layer Interferometry (BLI). hVISTA-ECD-Fc was incubated with a low-density anti-mFc-captured KVA12.2a mAb biosensor for a 240-second association period and transferred to PBS for a 360-second dissociation period. A 1:1 global curve fitting analysis was performed to determine equilibrium (KD), association (ka), and dissociation (kdis) rate constants. BLQ stands for below the limit of quantification.

hVISTA Mutations	KVA12.2a KD (M)	KVA12.2a Ka (1/Ms)	KVA12.2a Kdis (1/s)
None (WT)	< 1.0E-12	5.3E+05	3.3E-07
R54A, F62A, Q63A	4.1E-09	4.5E+05	1.9E-03
H66A, H68A, H72A, H93A, H94A, H121A, H122A, and H123A	< 1.0E-12	2.1E+06	5.1E-07
H121A, H122A, and H123A	< 1.0E-12	3.4E+06	6.0E-07
H66A, H68A	< 1.0E-12	2.3E+06	5.9E-07
N59A, T61A, D64A	2.5E-12	1.1E+06	2.7E-07
H79A, D80A, L81A	1.1E-12	3.8E+05	4.2E-07
Q83A, R84A, H85A	1.1E-12	3.9E+05	4.3E-07
T35A, I119A, S124A	< 1.0E-12	1.3E+06	4.7E-07
T35A	< 1.0E-12	2.9E+06	3.2E-07
I119A	< 1.0E-12	1.6E+06	3.8E-07
S124A	< 1.0E-12	3.5E+06	4.5E-07
T35A, I119A	< 1.0E-12	3.0E+06	4.0E-07
I119A, S124A	< 1.0E-12	3.3E+06	5.9E-07
T35A, S124A	< 1.0E-12	3.6E+06	5.3E-07
L115A, H129A	1.3E-12	2.2E+05	2.9E-07
Y37A, V117A, R127A	BLQ	BLQ	BLQ
Y37A	< 1.0E-12	1.2E+06	4.7E-07
V117A	< 1.0E-12	8.6E+06	3.9E-07
R127A	3.0E-10	1.1E+06	3.4E-04
Y37A, V117A	5.0E-11	9.0E+05	4.5E-05
V117A, R127A	1.7E-09	1.3E+06	2.2E-03
Y37A, R127A	2.7E-09	2.2E+06	6.0E-03
E47A, V48A, Q49A	< 1.0E-12	7.1E+05	3.9E-07
S52A, E53A, R55A	< 1.0E-12	8.0E+05	4.7E-07
Q73A, N76A	< 1.0E-12	5.2E+05	3.3E-07
H32A, D33A, T35A	< 1.0E-12	4.1E+05	2.7E-07

E14A, L106A, L107A	< 1.0E-12	5.5E+05	3.5E-07
N17Q, N59Q, N76Q, N96Q, N158Q	< 1.0E-12	6.9E+05	3.4E-07
N59Q, N76Q, N158Q	< 1.0E-12	9.2E+05	4.4E-07
N17Q, N96Q	< 1.0E-12	5.1E+05	2.9E-07
F36A	< 1.0E-12	7.4E+05	2.8E-07
K38A	< 1.0E-12	8.5E+05	4.9E-07
T39A	< 1.0E-12	1.0E+06	4.2E-07
E125A	2.3E-10	2.7E+06	6.2E-04
H122A	< 1.0E-12	5.4E+05	4.1E-07
R54A	BLQ	BLQ	BLQ
F62A	< 1.0E-12	5.2E+05	3.3E-07
Q63A	< 1.0E-12	4.6E+05	4.6E-07
R54A, R127A	BLQ	BLQ	BLQ
F62A, R127A	7.4E-09	6.6E+05	4.9E-03
Q63A, R127A	6.1E-09	4.4E+05	2.7E-03

Table S8. KVA12123 mAb shows reduced ADCC. KVA12123-mediated ADCC activity using human PBMCs and hVISTA expressing Raji cells. EC50s were calculated by using a four-parameter nonlinear regression fitting. The EC50 fold difference for KVA12123 and VSTB174 for six healthy donors is shown.

PBMC donor	VSTB174 EC50 (ug/mL)	KVA 12123 EC50 (ug/mL)	Fold difference
26349	0.010	0.050	4
11917	0.009	0.030	4
7347	0.007	0.010	1
14471	0.005	0.010	2
4058	0.010	0.010	1
18088	0.002	0.003	2
